# Biological Activity and Structure–Activity Relationship of Mono- and Bis-Derivatives of 3-(Arylamino)propanehydrazides

**DOI:** 10.3390/biom16070975

**Published:** 2026-07-02

**Authors:** Ingrida Tumosienė, Ilona Jonuškienė, Neringa Petrašauskienė, Kristina Kantminienė

**Affiliations:** 1 Department of Organic Chemistry, Kaunas University of Technology, Radvilėnų pl. 19, 50254 Kaunas, Lithuania; ingrida.tumosiene@ktu.lt (I.T.); ilona.jonuskiene@ktu.lt (I.J.); 2 Bioprocess Research Centre, Kaunas University of Technology, Radvilėnų pl. 19, 50254 Kaunas, Lithuania; 3 Department of Physical and Inorganic Chemistry, Kaunas University of Technology, Radvilėnų pl. 19, 50254 Kaunas, Lithuania; neringa.petrasauskiene@ktu.lt

**Keywords:** hydrazone, bioactive compounds, antioxidant activity, antibacterial activity, structure-activity relationship

## Abstract

A series of *N*’-(4-nitrobenzylidene)propanehydrazides and corresponding bishydrazones, as well as *N*-(1,3-dioxoisoindolin-2-yl)propanamides and corresponding bis(propanamides), were synthesised. Antioxidant properties were assessed using reducing power, FRAP, DPPH, ABTS radical scavenging, and NBT inhibition assays. Among the tested compounds, phenylaminopropanamide **17** demonstrated the highest activity, showing 1.70-fold higher reducing power and 6.71-fold higher DPPH radical scavenging activity compared to melatonin. Phenylaminopropanehydrazide **9** exhibited the highest activity in the FRAP assay (1.48 times higher than melatonin), while ethoxyphenyl-based bis(propanamide) **24** showed the greatest NBT inhibition (1.58-fold relative to melatonin). Four compounds exhibited moderate antibacterial activity against both Gram-positive and Gram-negative bacteria.

## 1. Introduction

The design and synthesis of novel small redox-active molecules that can modulate cellular oxidative processes remains an important strategy of modern chemical science [1,2]. Oxidative stress, which is defined as an imbalance between the production of reactive oxygen species (ROS) and the ability to neutralise them, is one of major areas of research in molecular science. This phenomenon is of particular importance due to its impact on cell homeostasis and damage to organic substrates. Furthermore, oxidative stress exerts a significant influence on microbial toxicity, showing a close correlation with the antibacterial activity of redox-active small molecules [3,4]. The development of antibiotic resistance may be determined by ROS generated as by-products of bacterial respiration and metabolism [5]. Consequently, the synthesis of novel compounds with potent antioxidant properties or the ability to act as controlled redox modulators is highly relevant to the development of new functional materials and biologically active scaffolds.

Hydrazones, which contain an azomethine linkage (-NH-N=CH-), constitute an important class of organic compounds due to their structural flexibility and varied reactivity profile [6,7,8]. This functional group not only ensures geometric flexibility of the molecule but, due to its electronic properties, participates in hydrogen bonding and can act as an electron donor or acceptor in redox reactions. Hydrazone derivatives possess a wide range of pharmacological properties, including antifungal [9,10,11], anticancer [12,13,14,15], and anti-inflammatory [7,16,17,18] effects. Several studies have demonstrated that hydrazone derivatives possess antioxidant [7,8,12,19] and antibacterial properties [10,20,21,22]. They are effective against Gram-positive bacteria, including *Staphylococcus aureus*, *Streptococcus pneumoniae*, and *Bacillus subtilis*, as well as Gram-negative strains such as *Escherichia coli*, *Klebsiella pneumoniae*, and *Pseudomonas aeruginosa* [22,23,24].

Bioactive hydrazone structures that incorporate nitroaromatic fragments, such as 4-nitrobenzylidene fragments, often exhibit strong antibacterial activity. This is due to their ability to participate in redox cycles, induce oxidative stress in microbial cells, and generate reactive intermediate metabolites [25]. The nitro group can be reduced in enzyme-catalysed reactions, and the intermediate products formed during these processes can damage bacterial nucleic acids, proteins, and membranes. The compounds under investigation have a molecular structure that combines redox-active nitroaromatic fragments, hydrazone bonds with multiple reactive centres, and electronically compatible aromatic amines. Nitroarylhydrazone derivatives obtained from nitroarylaldehydes and heteroarylhydrazines have shown antibacterial activity against sensitive and resistant bacterial strains. For example, 2-(4-nitrobenzylidene)-1-(quinazolin-4-yl)hydrazine has shown significant antimicrobial activity without cytotoxicity [26]. Furthermore, hydrazones containing 4-nitrobenzylidene moieties were evaluated for their ability to inhibit urease activity. For example, *N*′-(4-nitrobenzylidene)-2-(4-nitrophenoxy)acetohydrazide exhibited quantitatively measurable inhibition (IC_50_) values, suggesting their potential application in enzyme-based assays [27].

Isoindoline-1,3-dione derivatives represent a structurally relevant class of compounds possessing antibacterial and antioxidant properties [13,28]. Phthalimide derivatives have an inherent hydrophobic nature, indicated by the presence of the functional group (-CO-N(R)-CO) [13], which enhances their ability to traverse biological membranes within living organisms, a property crucial to achieving intracellular targets within bacterial cells. 2-[4-(1-Hydrazonoethyl)phenyl]isoindoline-1,3-dione exhibited significant antimicrobial activity against *Bacillus subtilis*, compared to standard antibiotics (ampicillin, cefotaxime, and gentamicin) [6]. Incorporation of the phthalimide moiety into arylamino-propanamide frameworks may enhance molecular stability and biological performance, thus providing an alternative to hydrazone-based systems.

Despite the extensive study of hydrazone and phthalimide derivatives individually, less attention has been given to systems that directly compare hydrazone-based structures with their amide-based analogues, particularly in relation to both antioxidant and antibacterial activities. Such comparisons are important for understanding how key functional groups influence redox behaviour and biological response.

**Figure 1:**
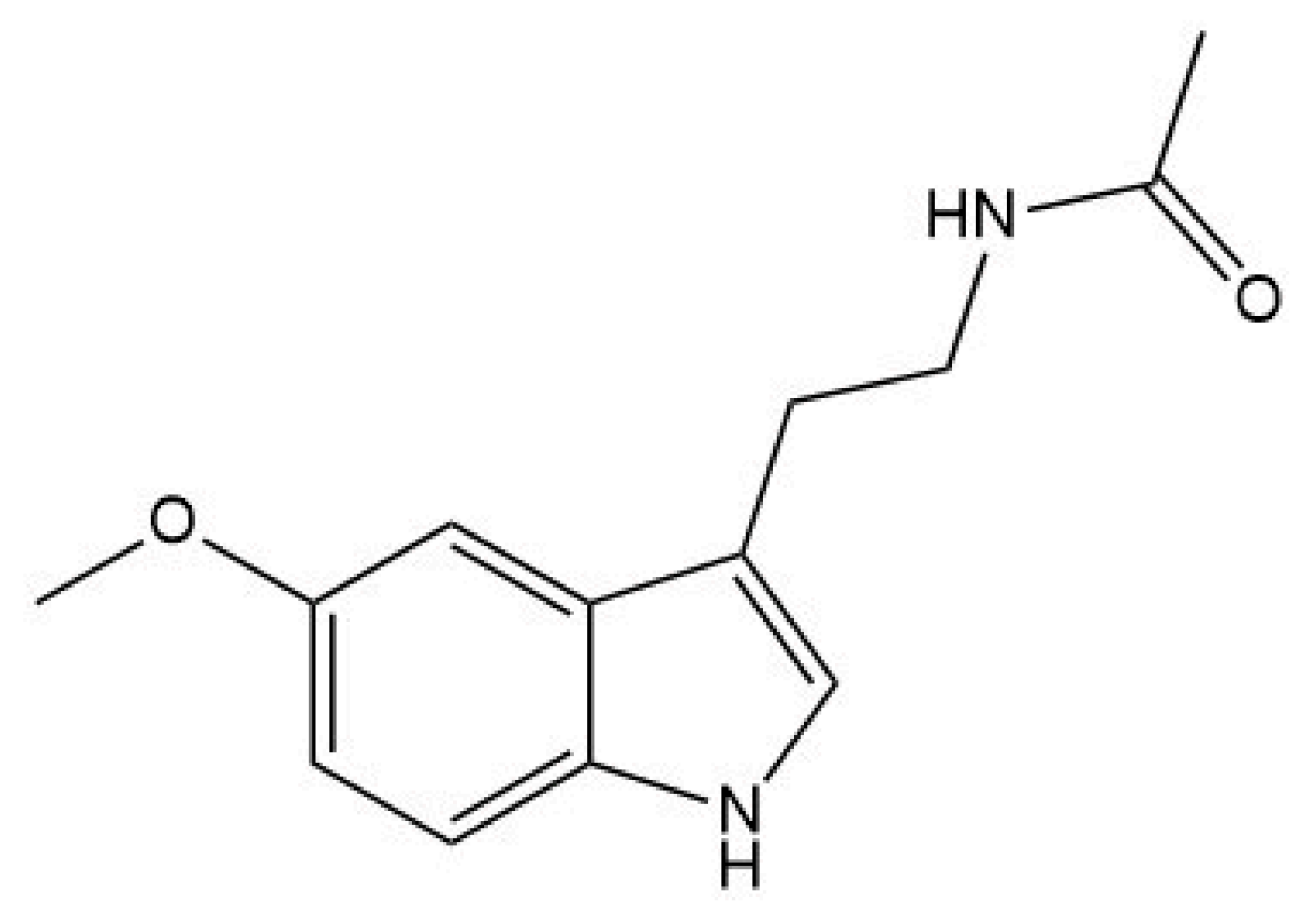
Structure of melatonin (*N*-acetyl-5-methoxytryptamine).

Melatonin (Figure 1) is a versatile molecule, which plays a significant role in regulating circadian rhythms, acting as an antioxidant and modulating immune responses. Its therapeutic potential spans a wide range of applications, from treating sleep disorders to supporting cancer therapy, which highlights its importance in both physiological and clinical contexts [29,30]. Melatonin is frequently used as a reference antioxidant due to its well-documented radical scavenging properties; however, its limited bioavailability and a short plasma half-life following oral administration [31] motivate the search for alternative compounds with improved performance.

Several indole-based hydrazone derivatives have been reported to exhibit stronger antioxidant effects in vitro than melatonin when evaluated using multiple oxidative stress-related assays [32,33,34,35].

The objective of this study was to design, synthesise, and comparatively evaluate 4-nitrobenzylidene hydrazone derivatives and structurally related phthalimide-containing propanamides, together with their bis-analogues, in terms of antioxidant and antibacterial activity using melatonin as a reference standard based on to the reported antioxidant and ROS scavenging properties of hydrazide- and hydrazone-derived compounds [36,37].

## 2. Materials and Methods

### 2.1. Synthesis

Reagents were purchased from Sigma-Aldrich (St. Louis, MO, USA) and TCI Europe N.V. (Zwijndrecht, Belgium). The reaction course and purity of the synthesised compounds were monitored by TLC using aluminium plates precoated with silica gel 60 F254 (MerckKGaA, Darmstadt, Germany). The melting points were determined on a MEL-TEMP (Electrothermal, A Bibby Scientific Company, Burlington, NJ, USA) melting point apparatus and are uncorrected. The ^1^H and ^13^C NMR spectra were recorded in DMSO-*d*_6_ on a Bruker Avance III (400 MHz, 101 MHz) spectrometer (Bruker BioSpin AG, Fällanden, Switzerland) operating in the Fourier transform mode. Chemical shifts (*δ*) are reported in parts per million (ppm) calibrated from TMS (0 ppm) as an internal standard for ^1^H NMR, and DMSO-*d*_6_ (39.43 ppm) for ^13^C NMR. In ^1^H NMR spectra, signal splitting is denoted using standard abbreviations: singlet (s), doublet (d), triplet (t), quartet (q), and multiplet (m). FT-IR spectra (ν, cm^−1^) were recorded on a Perkin–Elmer Spectrum BX FT–IR spectrometer (Perkin–Elmer Inc.,Waltham, MA, USA) using KBr pellets. Mass spectra were obtained on a Shimadzu LCMS-2050 liquid chromatograph–mass spectrometer (Shimadzu Corporation, Kyoto, Japan) with positive ESI ionisation. Elemental analyses (C, H, N) were performed using the CE-440 Elemental Analyzer (Exeter Analytical, Inc., North Chelmsford, MA, USA).

3,3′-(Phenylazanediyl)bis(*N*′-(4-nitrobenzylidene)propanehydrazide) (**13**) and 3,3′-(*p*-tolylazanediyl)bis(*N*′-(4-nitrobenzylidene)propanehydrazide) (**14**) were synthesised as described in [38]. 3,3′-((4-Methoxyphenyl)azanediyl)bis(*N*′-(4-nitrobenzylidene)propanehydrazide) (**15**) and 3,3′-((4-ethoxyphenyl)azanediyl)bis(*N*′-(4-nitrobenzylidene)propanehydrazide) (**16**) were synthesised as described in [39]. *N*-(1,3-dioxoisoindolin-2-yl)-3-((4-methoxyphenyl)amino)propanamide (**19**) was synthesised as described in [40]. 3,3′-(Phenylazanediyl)bis(*N*-(1,3-dioxoisoindolin-2-yl)propanamide) (**21**) and 3,3′-(*p*-tolylazanediyl)bis(*N*-(1,3-dioxoisoindolin-2-yl)propanamide) (**22**) were synthesised as described in [38]. 3,3′-((4-Methoxyphenyl)azanediyl)bis(*N*-(1,3-dioxoisoindolin-2-yl)propanamide) (**23**) and 3,3′-((4-ethoxyphenyl)azanediyl)bis(*N*-(1,3-dioxoisoindolin-2-yl)propanamide) (**24**) were synthesised as described in [39]. M.p., ^1^H (Figures S22–S30) and ^13^C NMR spectra were found to be identical with the reported ones [38,39,40].

General procedure for preparation of compounds **9**–**12**

A mixture of hydrazide **1**–**4** (3 mmol) and *p-*nitrobenzaldehyde (3.1 mmol) in methanol (30 mL) was heated at reflux for 10-30 min. The precipitate formed was filtered off, washed with diethyl ether, and recrystallized from DMF–water mixture.

*N′*-(4-nitrobenzylidene)-3-(phenylamino)propanehydrazide (**9**)

Prepared from 3-(phenylamino)propanehydrazide (**1**) [41] by heating the reaction mixture at reflux for 10 min. Yellow solid, yield 0.84 g (89%); m.p. 183–184 °C; IR (KBr) ν_max_ (cm^−1^): 3350, 2950 (NH), 1659 (C=O); ^1^H NMR (400 MHz, DMSO-*d*_6_): (*Z*/*E* isomeric mixture, 60/40): δ = 2.52 (t, 0.8H, *J* = 7.2 Hz, H_8_); 2.93 (t, 1.2H, *J* = 7.2 Hz, H_8_); 3.34 (q, 1.6H, *J* = 7.2 Hz, *J* = 12.8 Hz, H_7_); 5.62 (s, 1H, NH); 6.53 (t, 1H, *J* = 7.6 Hz, H_4_); 6.58 (d, 2H, *J* = 7.6 Hz, H_2,6_); 7.07 (t, 2H, *J* = 7.6 Hz, H_3,5_); 7.86 (d, 1.2H, *J* = 8.4 Hz, H_12,16_); 7.93 (d, 0.8H, *J* = 8.4 Hz, H_12,16_); 8.07 (s, 0.6H, H_10_); 8.24 (d, 2H, *J* = 8.0 Hz, H_13,15_); 8.28 (s, 0.4H, H_10_); 11.62 (s, 0.6H, NH); 11.73 (s, 0.4H, NH). ^13^C NMR (101 MHz, DMSO-*d*_6_): δ = 32.1, 34.3 (C_8_), 38.6 (C_7_), 112.2, 112.3 (C_2,6_), 115.9, 116.0 (C_3,5_), 124.2 (C_4_), 127.7, 128.1, 129.1, 140.7, 140.8, 143.8, 147.7, 147.9, 148.6, 148.7 (C_1,10–16_); 168.1; 173.7 (C_9_); MS (ESI^+^): *m*/*z* calculated for C_16_H_16_N_4_O_3_, 313 [M+H]^+^; found 313. Anal. calcd. for C_16_H_16_N_4_O_3_, %: C, 61.53; H, 5.16; N, 17.94. Found, %: C, 61.50; H, 5.14; N, 17.89.

*N′*-(4-nitrobenzylidene)-3-(*p*-tolylamino)propanehydrazide (**10**)

Prepared from 3-(*p*-tolylamino)propanehydrazide (**2**) [42] by heating the reaction mixture at reflux for 10 min. Orange solid, yield 0.83 g (84%); m.p. 201–202 °C; IR (KBr) ν_max_ (cm^−1^): 3347, 2966 (NH), 1664 (C=O); ^1^H NMR (400 MHz, DMSO-*d*_6_): (*Z*/*E* isomeric mixture, 60/40): δ = 2.13 (s, 3H, H_17_); 2.52 (t, 0.8H, *J* = 7.2 Hz, H_8_); 2.93 (t, 1.2H, *J* = 7.2 Hz, H_8_); 3.34 (q, 1.6H, *J* = 7.2 Hz, *J* = 12.8 Hz, H_7_); 5.36, 5.38 (2s, 1H, NH); 6.49 (d, 2H, *J* = 7.6 Hz, H_2,6_); 6.88 (t, 2H, *J* = 7.6 Hz, H_3,5_); 7.83 (d, 1.2H, *J* = 8.4 Hz, H_12,16_); 7.92 (d, 0.8H, *J* = 8.4 Hz, H_12,16_); 8.06 (s, 0.6H, H_10_); 8.21–8.25 (m, 2H, H_13,15_); 8.27 (s, 0.4H, H_10_); 11.61 (s, 0.6H, NH); 11.72 (s, 0.4H, NH); ^13^C NMR (101 MHz, DMSO-*d*_6_): δ = 32.1, 34.4 (C_8_); 39.0 (C_7_); 39.5 (C_17_), 112.4, 112.5 (C_2,6_); 124.2 (C_4_); 124.3, 124.5, 127.7, 128.1, 129.6, 140.7, 140.8, 143.7, 146.4, 147.7, 147.9, (C_1,3,5,10–16_), 168.1, 173.8 (C_9_); MS (ESI^+^): *m*/*z* calculated for C_17_H_18_N_4_O_3_, 327 [M+H]^+^; found 327. Anal. calcd.. for C_17_H_18_N_4_O_3_, %: C, 62.57; H, 5.56; N, 17.17. Found, %: C, 62.49; H, 5.60; N, 17.15.

3-((4-Methoxyphenyl)amino)-*N*′-(4-nitrobenzylidene)propanehydrazide (**11**)

Prepared from 3-((4-methoxyphenyl)amino)propanehydrazide (**3**) [43] by heating the reaction mixture at reflux for 20 min. Brown solid, yield 0.97 g (95%); m.p. 161–162 °C; IR (KBr) ν_max_ (cm^−1^): 3370, 2976 (NH), 1670 (C=O); ^1^H NMR (400 MHz, DMSO-*d*_6_): (*Z*/*E* isomeric mixture, 60/40): δ = 2.52 (t, 0.8H, *J* = 7.2 Hz, H_8_); 2.91 (t, 1.2H, *J* = 7.2 Hz, H_8_); 3.29 (q, 1.6H, *J* = 7.2 Hz, *J* = 12.8 Hz, H_7_); 3.62 (s, 3H, H_17_); 5.17 (s, 1H, NH); 6.53–6.56 (m, 2H, H_2,6_); 6.69–6.73 (m, 2H, H_3,5_); 7.83 (d, 1.2H, *J* = 8.4 Hz, H_12,16_); 7.92 (d, 0.8H, *J* = 8.4 Hz, H_12,16_); 8.05 (s, 0.6H, H_10_); 8.21–8.24 (m, 2H, H_13,15_); 8.26 (s, 0.4H, H_10_); 11.60 (s, 0.6H, NH); 11.72 (s, 0.4H, NH); ^13^C NMR (101 MHz, DMSO-*d*_6_): δ = 32.2, 34.5 (C_8_); 39.6 (C_7_); 40.1 (C_17_), 113.4, 113.5, 114.8 (C_2,6_); 124.2 (C_4_); 127.7, 128.1, 140.6, 140.7, 140.8, 142.9, 143.7, 147.7, 147.9, 150.9, 151.0 (C_1,3,5,10–16_), 168.2, 173.9 (C_9_); MS (ESI^+^): *m*/*z* calculated for C_17_H_18_N_4_O_4_, 343 [M+H]^+^; found 343. Anal. calcd. for C_17_H_18_N_4_O_4_, %: C, 59.64; H, 5.30; N, 16.37. Found, %: C, 59.62; H, 5.29; N, 16.30.

3-((4-Ethoxyphenyl)amino)-*N*′-(4-nitrobenzylidene)propanehydrazide (**12**)

Prepared from 3-((4-ethoxyphenyl)amino)propanehydrazide (**4**) [43] by heating the reaction mixture at reflux for 30 min. Yellow solid, yield 1.05 g (98%); m.p. 157–158 °C; IR (KBr) ν_max_ (cm^−1^): 3349, 2975 (NH), 1660 (C=O); ^1^H NMR (400 MHz, DMSO-*d*_6_): (*Z*/*E* isomeric mixture, 60/40): δ = 1.28 (t, 3H, *J* = 7.2 Hz, H_18_); 2.54 (t, 0.8H, *J* = 7.2 Hz, H_8_); 2.94 (t, 1.2H, *J* = 7.2 Hz, H_8_); 3.32 (t, 1.6H, *J* = 7.2 Hz, H_7_); 3.86–3.91 (q, 2H, *J* = 7.2 Hz, *J* = 12.8 Hz, H_17_); 5.23 (s, 1H, NH); 6.55–6.58 (m, 2H, H_2,6_); 6.71–6.74 (m, 2H, H_3,5_); 7.86 (d, 1.2H, *J* = 8.4 Hz, H_12,16_); 7.95 (d, 0.8H, *J* = 8.4 Hz, H_12,16_); 8.09 (s, 0.6H, H_10_); 8.24–8.27 (m, 2H, H_13,15_); 8.29 (s, 0.4H, H_10_); 11.64 (s, 0.6H, NH); 11.71 (s, 0.4H, NH); ^13^C NMR (101 MHz, DMSO-*d*_6_): δ = 14.9 (C_18_); 32.0, 34.4 (C_8_); 39.5, 40.1 (C_7_); 63.3 (C_17_), 113.2, 113.3, 115.4 (C_2,6_); 124.0 (C_4_); 127.5, 127.8, 140.3, 140.5, 140.6, 140.8, 142.7, 142.8, 143.4, 147.5, 147.7, 150.0 (C_1,3,5,10–16_), 167.9, 173.6 (C_9_); MS (ESI^+^): *m*/*z* calculated for C_18_H_20_N_4_O_4_, 357 [M+H]^+^; found 357. Anal. calcd. for C_18_H_20_N_4_O_4_, %: C, 60.66; H, 5.66; N, 15.72. Found, %: C, 60.55; H, 5.59; N, 15.70.

General procedure for preparation of compounds **17**, **18**, and **20**

To hydrazide **1**–**4** (3 mmol) dissolved in 1,4-dioxane (20 mL), phthalic anhydride (6 mmol) was added and the reaction mixture was heated at reflux for 4 h. Afterwards, Na_2_CO_3_ was added until pH 7. Precipitate formed was filtered off, washed with water, and recrystallized from methanol.

*N*-(1,3-dioxoisoindolin-2-yl)-3-(phenylamino)propanamide (**17**)

Prepared from 3-(phenylamino)propanehydrazide (**1**) [41]. White solid, yield 0.66 g (71%); m.p. 174–175 °C; IR (KBr) ν_max_ (cm^−1^): 3336, 3086 (NH), 1692, 1552 (C=O); ^1^H NMR (400 MHz, DMSO-*d*_6_): δ = 2.73 (t, 2H, *J* = 7.6 Hz, H_8_), 3.44 (t, 2H, *J* = 7.6 Hz, H_7_), 5.81 (s, 1H, NH), 6.99–7.34 (m, 5H, H_2-6_), 7.36–7.66 (m, 4H, H_12–15_), 11.28 (s, 1H, NH); ^13^C NMR (101 MHz, DMSO-*d*_6_): δ = 30.3 (C_8_), 44.2 (C_7_), 116.9, 120.1, 121.6, 124.8, 125.5, 128.6, 129.6, 129.9, 139.8 (C_1–6,11–16_), 162.8, 169.0, 172.4 (C_9,10,17_); MS (ESI^+^): *m*/*z* calculated for C_17_H_15_N_3_O_3_, 310 [M+H]^+^; found 310. Anal. calcd. for C_17_H_15_N_3_O_3_, %: C, 66.01; H, 4.89; N, 13.58. Found, %: C, 66.09; H, 4.81; N, 13.55.

*N*-(1,3-dioxoisoindolin-2-yl)-3-(*p*-tolylamino)propanamide (**18**)

Prepared from 3-(*p*-tolylamino)propanehydrazide (**2**) [42]. White solid, yield 0.73 g (75%); m.p. 179–180 °C; IR (KBr) ν_max_ (cm^−1^): 3341, 3026 (NH), 1642, 1519 (C=O); ^1^H NMR (400 MHz, DMSO-*d*_6_): δ = 2.13 (s, 3H, H_18_), 2.39 (t, 2H, *J* = 6.8 Hz, H_8_), 3.21 (q, 2H, *J* = 6.8 Hz, *J* = 13.2 Hz, H_7_), 5.26–5.29 (m, 1H, NH), 6.42–6.51 (m, 4H, H_2,3,5,6_), 6.86–6.90 (m, 4H, H_12-15_), 9.89 (s, 1H, NH); ^13^C NMR (101 MHz, DMSO-*d*_6_): δ = 20.2 (C_18_), 33.4 (C_8_), 36.2 (C_7_); 112.5, 119.3, 124.4, 124.6, 129.2, 129.6, 132.2, 136.8, 146.4, 146.5, 152.7 (C_1–6,11–-16_), 169.9, 170.1, 171.2 (C_9,10,17_); MS (ESI^+^): *m/z* calculated for C_18_H_17_N_3_O_3_, 324 [M+H]^+^; found 324. Anal. calcd. for C_18_H_17_N_3_O_3_, %: C, 66.86; H, 5.30; N, 13.00. Found, %: C, 66.82; H, 5.29; N, 13.01.

*N*-(1,3-dioxoisoindolin-2-yl)-3-((4-ethoxyphenyl)amino)propanamide (**20**)

Prepared from 3-((4-ethoxyphenyl)amino)propanehydrazide (**4**) [43]. White solid, yield 0.90 g (85%); m.p. 191–192 °C; IR (KBr) ν_max_ (cm^−1^): 3337, 2980 (NH), 1640, 1509 (C=O); ^1^H NMR (400 MHz, DMSO-*d*_6_): δ = 1.25 (t, *J* = 6.8 Hz, 3H, H_19_), 2.38 (t, *J* = 6.8 Hz, 2H, H_8_), 3.19 (t, 2H, *J* = 6.8 Hz, H_7_), 3.84–3.89 (m, 2H, H_18_), 5.06 (s, 1H, NH), 6.52 (d, *J* = 7.2 Hz, 2H, H_2,6_), 6.70 (d, *J* = 7.2 Hz, 2H, H_3,5_), 6.89–7.13 (m, 4H, H_12–15_), 9.87 (s, 1H, NH); ^13^C NMR (101 MHz, DMSO-*d*_6_): δ = 15.3 (C_19_), 33.7 (C_8_), 40.7 (C_7_); 63.8 (C_18_), 113.7, 113.9, 115.9, 127.9, 131.0, 143.1, 150.6 (C_1–6,11–-16_), 161.8, 170.6 (C_9,10,17_); MS (ESI^+^): *m*/*z* calculated for C_19_H_19_N_3_O_4_, 354 [M+H]^+^; found 354. Anal. calcd. for C_19_H_19_N_3_O_4_, %: C, 64.58; H, 5.42; N, 11.89. Found, %: C, 64.56; H, 5.39; N, 11.83.

3,3′-(Phenylazanediyl)bis(*N*-(1,3-dioxoisoindolin-2-yl)propanamide) (**21**) was synthesised as described in [38]. M.p., ^1^H and ^13^C NMR spectra were found to be identical with the ones described in [38].

3,3′-(*p*-tolylazanediyl)bis(*N*-(1,3-dioxoisoindolin-2-yl)propanamide) (**22**) was synthesised as described in [38]. M.p., ^1^H and ^13^C NMR spectra were found to be identical with the ones described in [38].

3,3′-((4-Methoxyphenyl)azanediyl)bis(*N*-(1,3-dioxoisoindolin-2-yl)propanamide) (**23**) was synthesised as described in [39]. M.p., ^1^H and ^13^C NMR spectra were found to be identical with the ones described in [39].

3,3′-((4-Ethoxyphenyl)azanediyl)bis(*N*-(1,3-dioxoisoindolin-2-yl)propanamide) (**24**) was synthesised as described in [39]. M.p., ^1^H and ^13^C NMR spectra were found to be identical with the ones described in [39].

### 2.2. Determination of the Antioxidant Activity

#### 2.2.1. Ferric Ion (Fe^3+)^ Reducing Antioxidant Power Determination Assay

A solution (0.5 mL) of each compound in DMSO (20 mM) was mixed with phosphate buffer (1.25 mL, 0.2 M, pH 6.6) and potassium ferricyanide K_3_[Fe(CN)_6_] (1.25 mL, 1%). The mixture was incubated at 50 °C for 20 min. Aliquots (1.25 mL) of trichloroacetic acid (10%) were added to the mixture, which was then centrifuged for 10 min at 9000 rpm using a Hettich Universal 320 centrifuge (Hettich GmbH & Co., Tuttlingen, Germany). The upper layer of solution (1.25 mL) was combined with distilled water (1.25 mL) and ferric chloride (FeCl_3_, 0.25 mL, 0.1%). The absorbance of the samples was recorded at 700 nm (UV-1280 spectrophotometer (Shimadzu Corporation, Kyoto, Japan)) [44,45]. Melatonin was used as a positive control. All experiments were performed in triplicate.

#### 2.2.2. Ferric Reducing Antioxidant Power (FRAP) Assay

The FRAP reagent contained 2.5 mL of 10 mM TPTZ (2,4,6-tripyridyl-*s*-triazine) solution in 40 mM HCl, 2.5 mL of FeCl_3_ (20 mM) and 25 mL of acetate buffer (0.3 M, pH 3.6) [46,47]. The samples (50 µL) of the analysed compounds in DMSO (20 mM) were mixed with 1.5 mL of the FRAP reagent. The absorbance of the reaction mixture was measured spectrophotometrically (UV-1280 spectrophotometer (Shimadzu Corporation, Kyoto, Japan)) at 593 nm. For the calibration curve [48], five concentrations of FeSO_4_ · 7H_2_O (5, 10, 15, 20, 25 μM) were used. Melatonin was used as a positive control. All experiments were performed in triplicate.

#### 2.2.3. 1,1-Diphenyl-2-picrylhydrazyl (DPPH) Radical Scavenging Assay

The free radical scavenging activity of compounds was measured by the DPPH method [49,50]. First, a solution (20 mM) of each compound in DMSO was prepared. Subsequently, a 0.1 mM solution of DPPH in ethanol was prepared and 1 mL of this solution was added to the solutions of the analysed samples. The mixture was stirred vigorously and allowed to stand at room temperature for 20 min. The absorbance of the reaction mixture was measured at 517 nm (UV-1280 spectrophotometer (Shimadzu Corporation, Kyoto, Japan)). Melatonin was used as a positive control. All experiments were performed in triplicate. Antioxidant activity is expressed as the radical scavenging activity calculated according to the following formula:

(1) $$Radical\;scavenging\;activity\left(\%\right)=\frac{A_{c}-A_{s}}{A_{c}}\times 100,$$

where *A_c_* is the absorbance of the control sample and *A_s_* is the absorbance of the analysed compounds.

#### 2.2.4. 2,2-Azino-bis(3-ethylbenzothiazoline-6-sulfonic Acid) (ABTS) Radical Scavenging Assay

The ABTS stock solution (2 mM) was prepared by reacting ABTS with 0.17 mM potassium persulfate in 20 mM phosphate buffer (pH = 7.4) at room temperature under dark conditions for 12 h [51]. A working ABTS solution was then prepared by diluting the stock solution with 20 mM phosphate buffer (pH = 7.4). The working reagent was adjusted to give an absorbance of 1.5 ± 0.05 at 734 nm. Then, 0.5 mL of solution (20 mM) of the tested compound in DMSO was reacted with 0.3 mL of the working ABTS solution and 1.7 mL of 20 mM phosphate buffer (pH = 7.4). The ABTS radical scavenging ability was measured at 734 nm wavelengths using the UV/Visible spectrophotometer UV-1280 (Shimadzu Corporation, Kyoto, Japan). The radical scavenging activity (%) was calculated using formula (1). Melatonin was used as a positive control. All experiments were performed in triplicate.

#### 2.2.5. Nitroblue Tetrazolium (NBT) Inhibition

Nitroblue tetrazolium was used for the determination of superoxide anion radical ($O_{2}^{-}$) inhibition [52]. The reaction mixture contained 40 µL of the analysed compound (20 mM) in DMSO, 400 µL of 200 mM Tris-HCl buffer (pH = 7.8), 200 µL of 100 mM *L*-methionine, 200 µL of 540 µM nitroblue tetrazolium, 500 µL of 0.1% Triton X-100, 20 µL of 300 µM riboflavin and 640 µL of distilled water. A control reaction was performed without the compound sample. The reaction was carried out by exposing the reaction mixture to fluorescent light for 30 min at room temperature. After 30 min incubation, absorbance was recorded at 560 nm using a UV-1280 UV/Visible spectrophotometer (Shimadzu Corporation, Kyoto, Japan). NBT inhibition (%) was calculated using formula (1). Melatonin was used as a positive control. All experiments were performed in triplicate.

### 2.3. Determination of the Antibacterial Activity

Antibacterial activity of the compounds was screened by the disc diffusion method [53]. In this study, inhibition of bacterial growth was investigated against Gram-positive bacteria *Bacillus subtilis* and Gram-negative bacteria *Escherichia coli*. The solution (20 mM) of each screened compound was prepared in DMSO. Bacterial cultures were cultivated in *Petri* dishes at 37 °C for 24 h on the Luria–Bertani (LB) agar medium. Then, 50 µL of inoculum containing bacterial cells were spread across the LB agar medium. Sterile filter-paper discs were soaked in 25 µL of each compound solution, and then the discs were placed on the LB agar medium. Ciprofloxacin (20 mM) was used as a positive control, and DMSO was used as a negative control. *Petri* dishes were incubated aerobically at 37 °C and examined for zones of inhibition after 24 h. The inhibition zones (cm) were measured. All experiments were performed in triplicate.

### 2.4. Statistical Analysis

An unpaired two-tailed *t*-test was used to carry out statistically significant comparisons between melatonin (antioxidant activity) and ciprofloxacin (antibacterial activity), with a significance level set at *p* < 0.01. The analysis was performed using GraphPad Prism version 8.0.2 for Windows (San Diego, CA, USA).

## 3. Results and Discussion

### 3.1. Synthesis

**Scheme 1:**
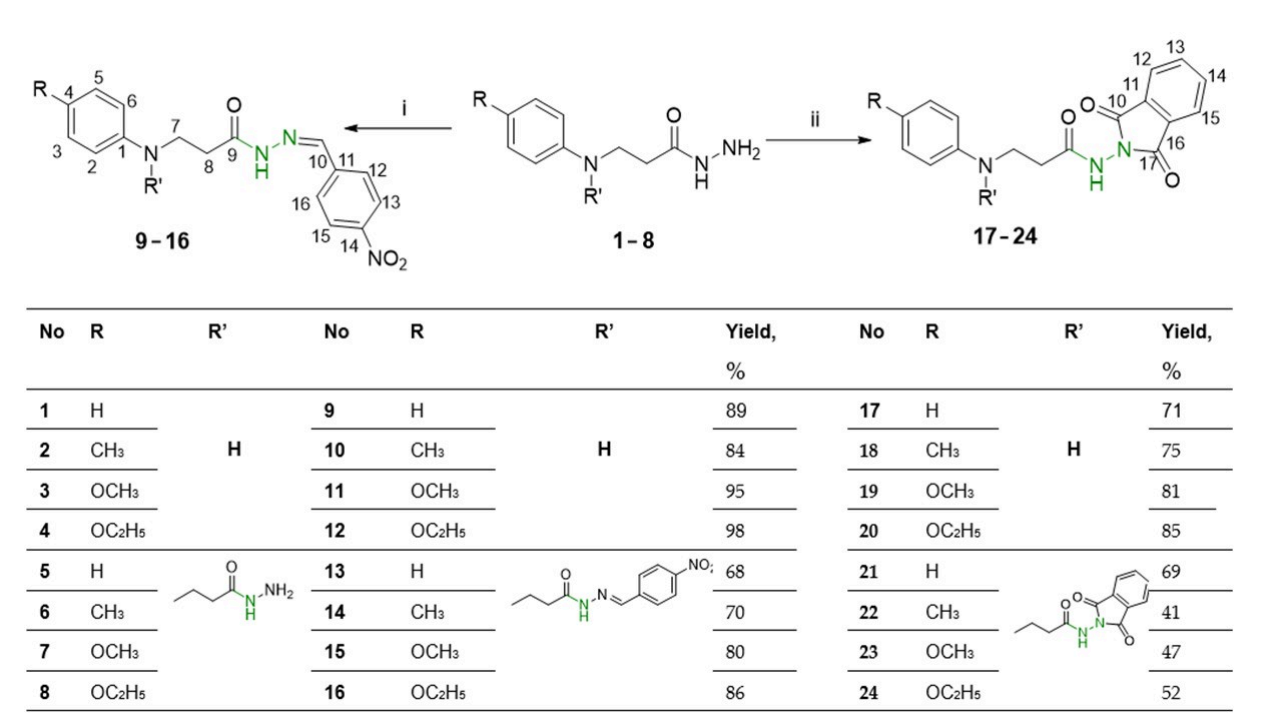
Synthesis of target compounds **9**–**24**. Reaction conditions: (i) *p*-nitrobenzaldehyde (at 1:1.1 ratio for **9**–**12** and 1:4 ratio for **13**–**16**), MeOH, reflux, 10–30 min; (ii) phthalic anhydride (at 1:2 ratio for **17**–**20** and 1:4 ratio for **21**–**24**), 1,4-dioxane, reflux, 4 h.

Compounds containing two identical pharmacophoric units often show enhanced biological activity compared to their analogues bearing a single set of these scaffolds. A series of sixteen target compounds, **9**–**24**, bearing one or two sets of propanehydrazide/propanamide moieties, were synthesised following the synthetic route outlined in Scheme 1. Hydrazone derivatives **9**–**12** were synthesised from 3-(phenylamino)propanehydrazide (**1**) [41] or corresponding 3-(4-substituted phenylamino)propanehydrazide **2**–**4** [42,43] and *p-*nitrobenzaldehyde at a 1:1.1 ratio in methanol under reflux with 84–98% yield. Bis(*N*’-(4-nitrobenzylidene)propanehydrazides) **13**–**16** were resynthesized through the reaction of the corresponding dihydrazides **5**–**8** and *p-*nitrobenzaldehyde at a 1:4 ratio as reported in [38,39]. Reaction of hydrazides **1**–**4** with phthalic anhydride at a 1:2 ratio in 1,4-dioxane provided corresponding phthaloyl hydrazides **17**–**20**. Unreacted phthalic anhydrides were hydrolyzed with aqueous Na_2_CO_3_ solution. Bis(*N*-(1,3-dioxoisoindolin-2-yl)propanamides) **21**–**24** were obtained through the reaction of dihydrazides **5**–**8** and phthalic anhydride at a 1:4 ratio following the synthesis procedure reported in [38,39].

The structures of the synthesised compounds were confirmed by ^1^H NMR, ^13^C NMR, FT-IR and MS data. In the ^1^H NMR spectra of monohydrazones **9**–**12**, the singlet in the range of 8.05–8.30 ppm is attributed to the CH proton of the benzylidene moiety. The splitting of the resonances of this proton, as well as those of the NH group proton and aromatic protons in 4-nitrophenyl ring, is due to the existence of hydrazones as a mixture of *Z*/*E* isomers resulting from the hindered rotation around the amide bond in DMSO-*d*_6_ solutions. The relative abundance of these isomers is influenced by several factors, including the electronic nature of substituents, solvent polarity, and temperature. The presence of both isomers in solution leads to distinct sets of signals corresponding to each configuration. NMR spectroscopy provides a useful tool for distinguishing between the two configurations, as the *Z* and *E* isomers typically exhibit distinct NH proton chemical shifts. The relative intensities of these signals (60:40 in the case of compounds **9**–**12**) suggest that the Z/E ratio is influenced by intramolecular hydrogen bonding and solvent effects in DMSO-*d*_6_. In general, the NH resonance of the *E* isomer is shifted downfield compared to that of the *Z* isomer, reflecting differences in the strength and orientation of intramolecular hydrogen-bonding interactions [54]. The carbonyl group carbon resonated in the range of 173.5–174 ppm in the ^13^C NMR spectra of **9**–**12**. In the ^13^C NMR spectra of phthaloyl hydrazides **17**, **18**, and **20**, carbons of three carbonyl groups gave signals in the range of 161.8–172.4 ppm. In the NMR spectra of bis-derivatives **13**–**16** and **21**–**24,** the double intensity of the resonances occurring due to the presence of double hydrazone moieties, substituted benzene rings or isoindole-1,3-dione has proven the structures of the target compounds [38,39]. The formation of the bis-derivatives was additionally confirmed by mass spectrometry, which showed molecular ion peaks corresponding to the expected molecular masses.

### 3.2. Evaluation of Biological Activity

Nonphenolic hydrazones exhibit significant antioxidant activity, driven by their structural features and electron-donating capabilities. Their effectiveness in radical scavenging highlights their potential for therapeutic applications, warranting further investigation into their mechanisms and optimisation. Hydrazone derivatives have been widely reported to exhibit antioxidant activity in various radical scavenging assays. These molecules are commonly thought to exhibit activity through alternative mechanisms, such as the donation of protons from nitrogen-containing functional groups (particularly the NH proton of the azomethine group) and the stabilisation of resulting radicals through conjugation and resonance within the aromatic system. Furthermore, the electronic and steric effects of substituents on the aromatic ring can have a significant influence on antioxidant activity, with electron-donating groups enhancing the capacity for radical scavenging [55,56].

**Figure 2:**
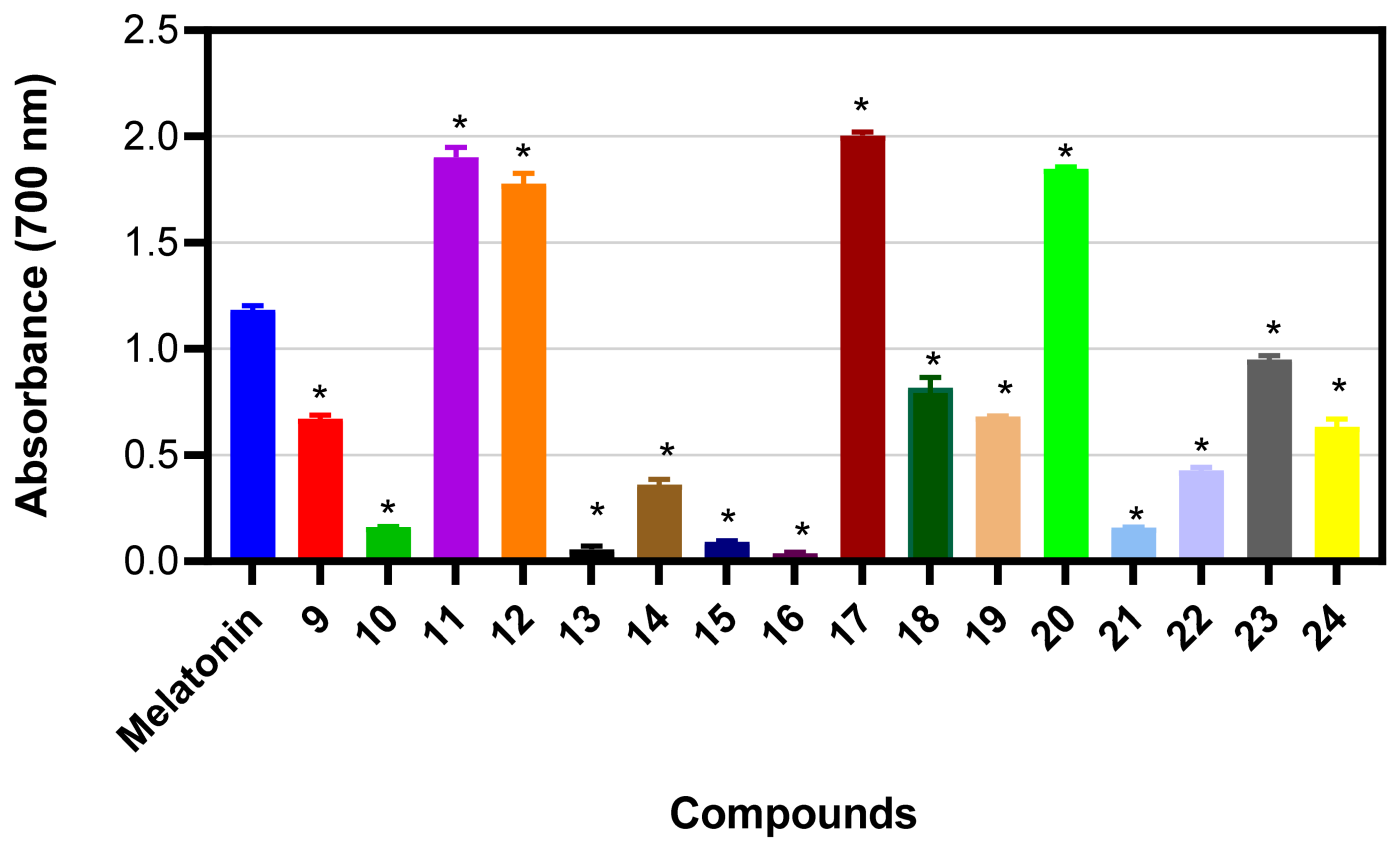
Antioxidative activity according to the reducing power assay of compounds **9**–**24** (20 mM). The error bars show the mean ± SD from three replicates. Asterisks show statistically significant comparisons between melatonin and compounds that were tested with an unpaired two-tailed *t*-test, with a significance level set at * *p* < 0.01.

Reducing power assay. As seen from the results presented in Figure 2, the reducing power activity of *N*-(1,3-dioxoisoindolin-2-yl)-3-(phenylamino)propanamide (**17**) was the highest (2.005 o.u. ± 0.017) and was 1.70 times (*p* < 0.01) higher than that of melatonin (1.18 o.u. ± 0.020).

Compounds **11** (1.902 o.u. ± 0.046), **20** (1.850 o.u. ± 0.007), and **12** (1.779 o.u. ± 0.046) were also more active than the control. It is interesting to note that despite the expectation for the increased potency of the compounds bearing dual-pharmacophores, monopropanehydrazides and monopropanamides were shown to possess higher antioxidant activity according to this assay. Between two 4-ethoxyphenyl-derived monosubstituted derivatives—monohydrazone **12** and monopropanamide **20**—the one bearing 1,3-dioxoisoindolinyl moiety possessed higher antioxidant activity.

**Figure 3:**
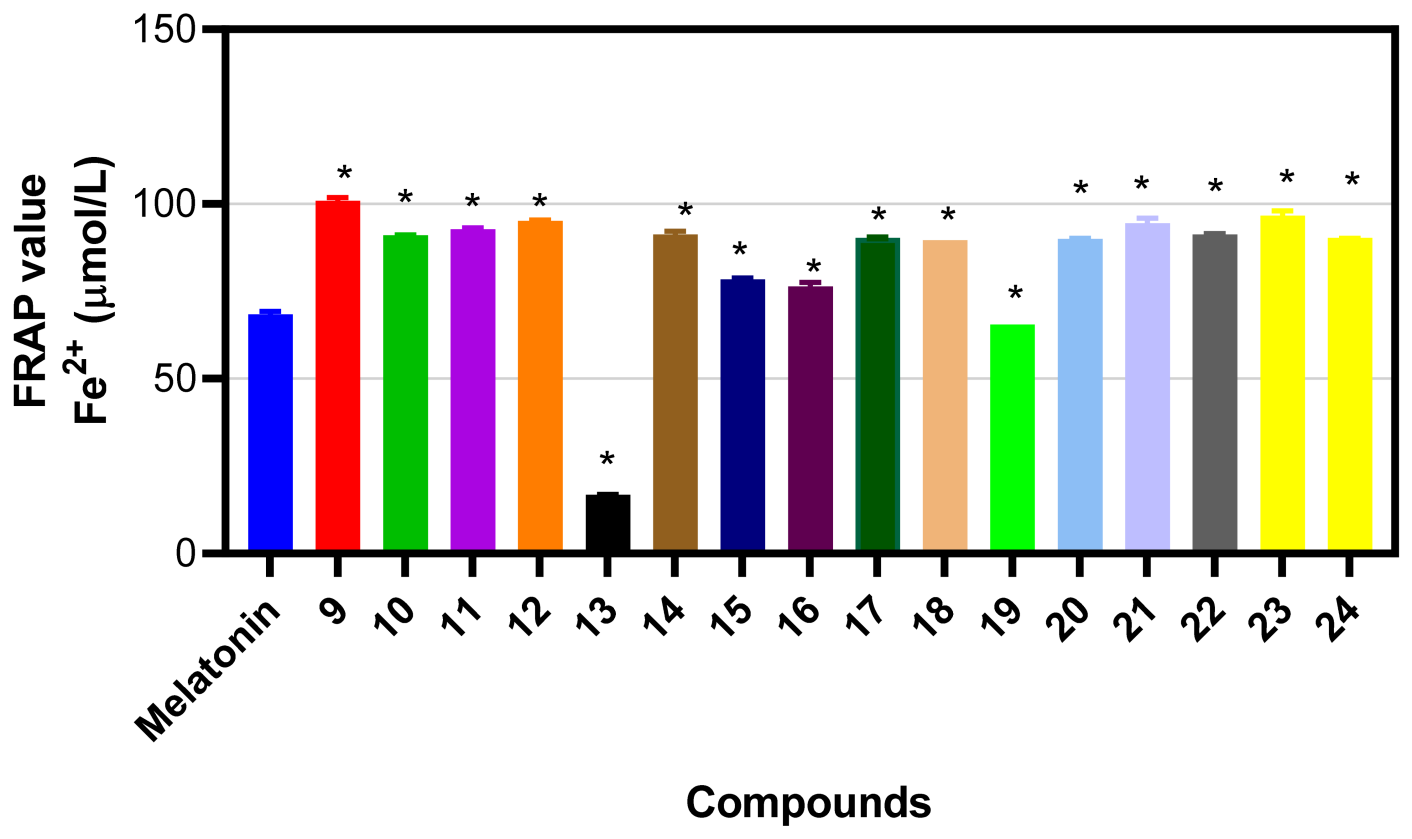
Antioxidative activity according to the FRAP assay of compounds **9**–**24** (20 mM). The error bars show the mean ± SD from three replicates. Asterisks show statistically significant comparisons between melatonin and compounds that were tested with an unpaired two-tailed *t*-test, with a significance level set at * *p* < 0.01.

The ferric reducing antioxidant power (FRAP) assay. Almost all tested compounds exhibited higher antioxidant activity than melatonin (68.40 µmol/L ± 0.846) (Figure 3).

*N*′-(4-nitrobenzylidene)-3-(phenylamino)propanehydrazide (**9**) was shown to possess the highest antioxidant activity (100.89 µmol/L ± 0.959), which was 1.48 (*p* < 0.01) times higher than that of melatonin. The unsubstituted phenylamine-derived bishydrazone **13** turned out to be the least active compound among all tested ones. In the pairs of 4-alkoxyphenylamine-derived hydrazones, monohydrazones **12** (95.23 µmol/L ± 0.212) and **11** (92.86 µmol/L ± 0.261) possessed higher antioxidant activity than bishydrazones **16** (76.48 µmol/L ± 0.261) and **15** (78.47 µmol/L ± 0.287), respectively. However, in the case of the least active electron-donating substituent of the phenyl ring (methyl group), bishydrazone **14** (91.36 µmol/L ± 0.861) was shown to be more active than monohydrazone **10** (91.02 µmol/L ± 0.165). In the pair of phenylamine-derived mono- and bispropanamides, bearing the 1,3-dioxoisoindolinyl moiety, bispropanamide **21** (94.51 µmol/L) was more active than monopropanamide **17** (90.27 µmol/L ± 0.241). The antioxidant activity of 4-methoxyphenylamine-derived bispropanamide **23** (96.73 µmol/L ± 1.340) was confirmed to be the second best among the evaluated compounds, while propanamide **19** (65.51 µmol/L ± 0.007) was the second least active compound. The replacement of the methoxy group with the slightly less active electron-donating ethoxy group followed the pattern suggesting that bispropanamide is more active than monopropanamide (**24** (90.33 µmol/L ± 0.215) vs.** 20** (90 µmol/L ± 0.141)). The same pattern was observed for the antioxidant activity of 4-methylphenylamine-derived bis- and monopropanamides (**22** (91.35 µmol/L ± 0.267) vs. **18** (89.70 µmol/L ± 0.027)). Nonphenolic hydrazones exhibited antioxidant properties through mechanisms like radical scavenging via the imine (C=N) group and electron donation from substituents. Electron-donating groups such as -CH_3_, -OCH_3_ or -OCH_2_CH_3_ on the aromatic rings enhanced this activity by increasing electron density, facilitating hydrogen atom transfer (HAT) or single-electron transfer (SET) to radicals [57]. When comparing the antioxidant activity according to FRAP assay among 4-nitrobenzylidenepropanehydrazides and corresponding *N*-(1,3-dioxoisoindolin-2-yl)propanamides, it should be noted that monohydrazones are more active than monopropanamides (**9** vs. **17**; **10** vs. **18**; **12** vs. **20**), while, in the majority of cases, bispropanamides are more potent antioxidants than bishydrazones (**21** vs. **13**; **23** vs. **15**; **24** vs. **16**).

The 2,2-diphenyl-1-picrylhydrazyl (DPPH) assay. Hydrazones scavenge DPPH radicals primarily through HAT from the hydrazone NH group and SET facilitated by the electron-rich imine (C=N) moiety. The HAT mechanism involves direct abstraction of the labile NH hydrogen by the DPPH radical, forming a stable hydrazone radical delocalized across the C=N-N system and yielding DPPH-H (colourless) [58]. SET proceeds via electron donation from the hydrazone’s highest occupied molecular orbital (HOMO) and is often enhanced by electron-donating substituents, reducing DPPH- to its anion; a subsequent proton transfer completes scavenging [45].

**Figure 4:**
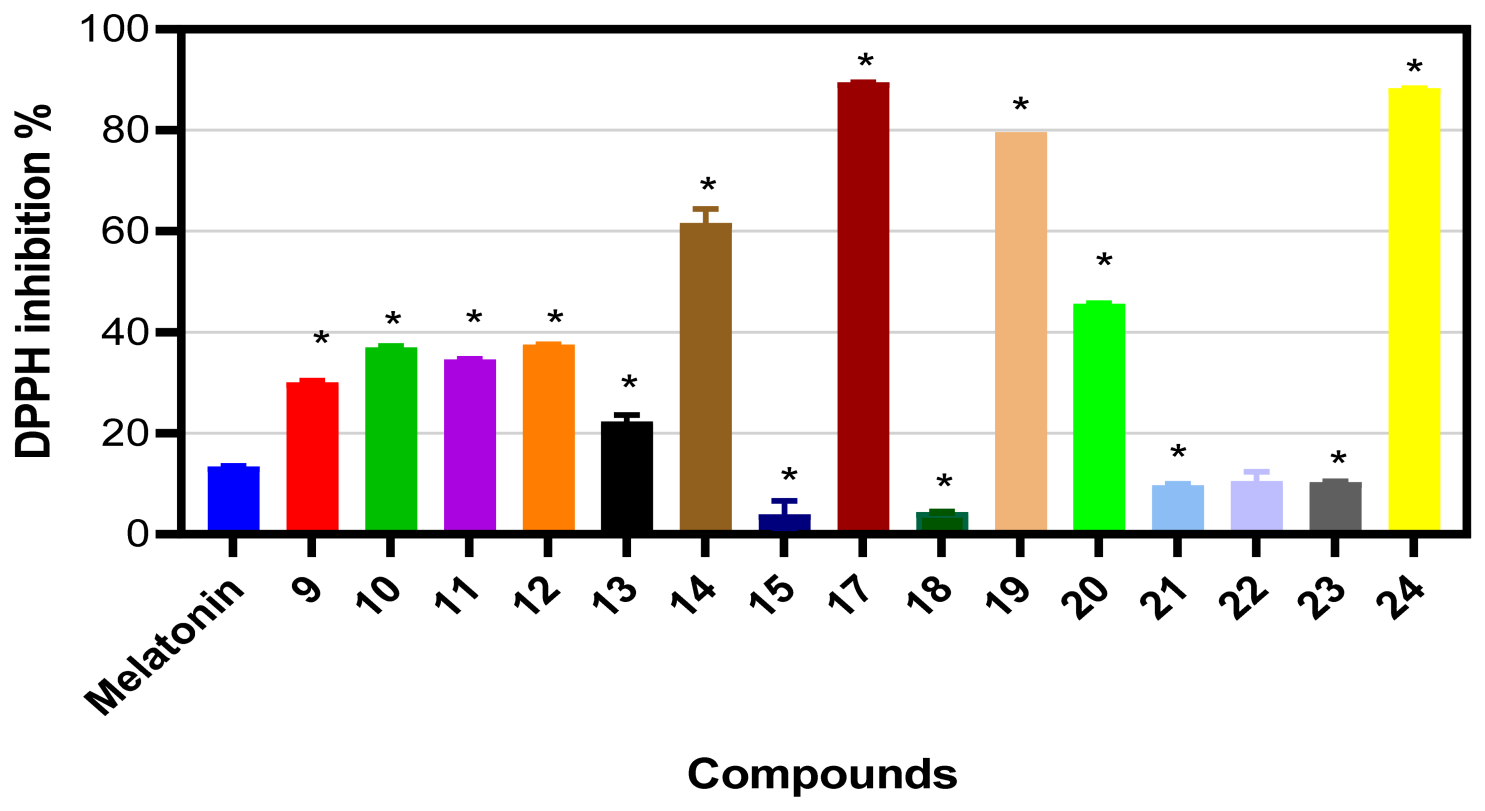
Antioxidative activity according to the DPPH assay of compounds **9**–**15** and **17**–**24** (20 mM; only compounds whose antioxidant activity could be measured under the experimental conditions are shown). The error bars show the mean ± SD from three replicates. Asterisks show statistically significant comparisons between melatonin and compounds that were tested with an unpaired two-tailed *t*-test, with a significance level set at * *p* < 0.01.

Among the tested compounds (Figure 4), eleven compounds were identified as possessing higher antioxidant activity than melatonin (13.33% ± 0.185). Likewise, as tested by the power-reducing assay, *N*-(1,3-dioxoisoindolin-2-yl)-3-(phenylamino)propanamide (**17**) (89.43% ± 0.092) was shown to be the most active one, surpassing the activity of the positive control 6.71 (*p* < 0.01) times. This finding is consistent with the results of other studies [34,59]. The antioxidant activity of 4-ethoxyphenylamine-derived bispropanamide **24** (88.31% ± 0.092) was just slightly lower, followed by 4-methoxy- and 4-ethoxyphenylamine-derived propanamides **19** (79.62% ± 0.006) and **20** (45.59% ± 0.244), respectively. The antioxidant performance followed the general trend methoxy ≳ ethoxy > methyl [60,61]. 4-Nitrobenzylidenepropanehydrazides were shown to be less active than respective *N*-(1,3-dioxoisoindolin-2-yl)propanamides. Antioxidant activity according to the DPPH assay of 3,3′-(*p*-tolylazanediyl)bis(*N*′-(4-nitrobenzylidene)propanehydrazide) (**14**) (61.64% ± 2.807) was shown to be the highest among the screened hydrazones. It should be noted that majority of other active hydrazones are monoderivatives (**12** (37.56% ± 0.092, 4-ethoxyphenylamine derivative), **10** (36.93%, 4-methylphenylamine derivative), **11** (34.64% ± 0.16 4-methoxyphenylamine derivative), and **9** (30.07% ± 0.403), unsubstituted phenylamine derivative).

**Figure 5:**
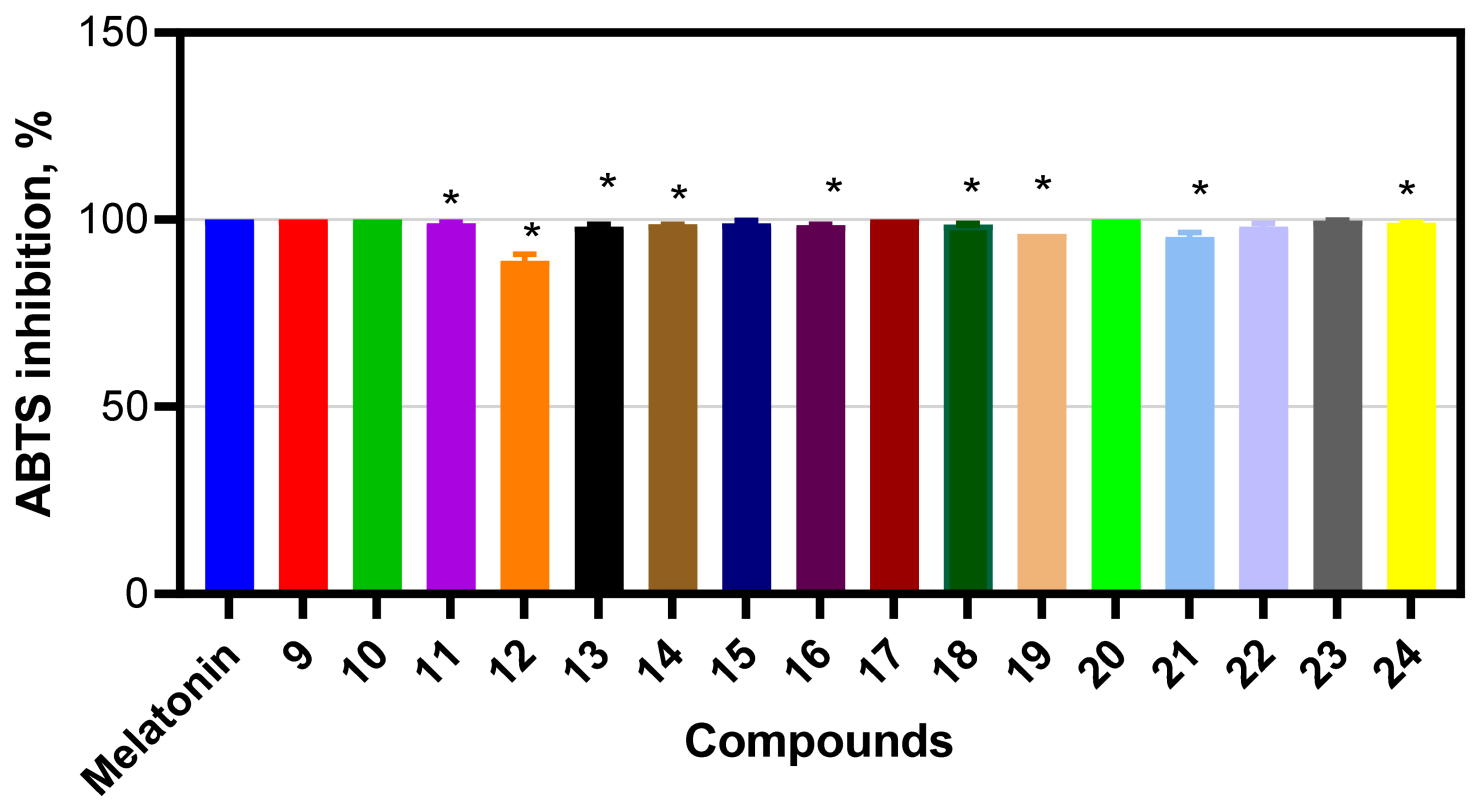
Antioxidative activity according to the ABTS assay of compounds **9**–**24** (20 mM). The error bars show the mean ± SD from three replicates. Asterisks show statistically significant comparisons between melatonin and compounds that were tested with an unpaired two-tailed *t*-test, with a significance level set at * *p* < 0.01.

The 2,2′-azino-bis(3-ethylbenzothiazoline-6-sulfonic acid (ABTS) assay. Among the synthesised compounds, four compounds exhibited the same maximal antioxidant activity as melatonin (100%) according to the ABTS assay (Figure 5).

*N*′-(4-nitrobenzylidene)-3-(phenylamino)propanehydrazide (**9**) (100%) and the corresponding propanamide **17** (100%) were among them. 4-Methylphenylamine-based hydrazone **10** (100%) and 4-ethoxyphenylamine-based propanamide **20** (100%) were the other two compounds with on par activity as the positive control. It should be noted that other screened compounds also showed very high activity according to ABTS assay.

**Figure 6:**
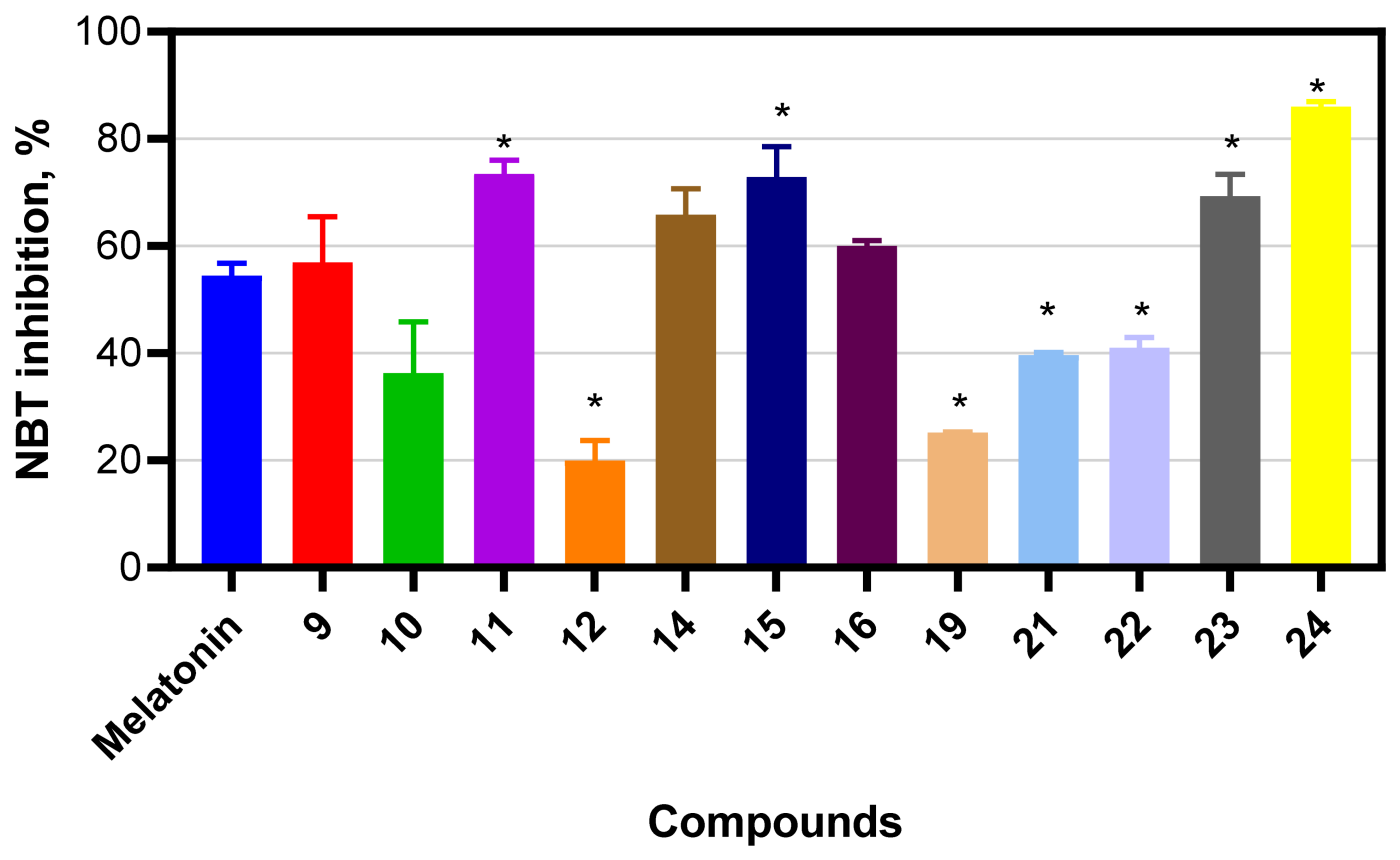
NBT inhibition of compounds **9**–**12**, **14**–**16**, **19**, and **21**–**24** (20 mM; only compounds whose antioxidant activity could be measured under the experimental conditions are shown). The error bars show the mean ± SD from three replicates. Asterisks show statistically significant comparisons between melatonin and compounds that were tested with an unpaired two-tailed *t*-test, with a significance level set at * *p* < 0.01.

The nitroblue tetrazolium (NBT) reduction method. The NBT assay turned out to be more selective than the ABTS assay, allowing a wider distribution of antioxidant activity among the screened compounds (Figure 6).

3,3′-((4-Ethoxyphenyl)azanediyl)bis(*N*-(1,3-dioxoisoindolin-2-yl)propanamide) (**24**) (86.02% ± 0.950) was shown to possess the highest NBT inhibition, which was 1.58 (*p* < 0.01) times higher than that of melatonin (54.43% ± 2.368), while the corresponding bispropanehydrazide **16** (60.04%) exhibited much lower activity. NBT inhibition values of 4-methoxyphenylamine-based monohydrazone **11** (73.42% ± 2.580) and bishydrazone **15** (72.83% ± 5.710) were very similar, with the monoderivative being just slightly more active than its bis-counterpart. Replacement of the methoxy group (in **15**) with a slightly less active electron-donating ethoxy group decreased the antioxidant activity of **16** (60.04% ± 1.034) to follow the general trend of methoxy group-bearing compounds exhibiting higher antioxidant activity than their ethoxy analogues [60,61]. According to the NBT assay bis(*N*-(1,3-dioxoisoindolin-2-yl)propanamide) **23** (69.29% ± 4.101) is less active than the corresponding bishydrazone **15**.

**Figure 7:**
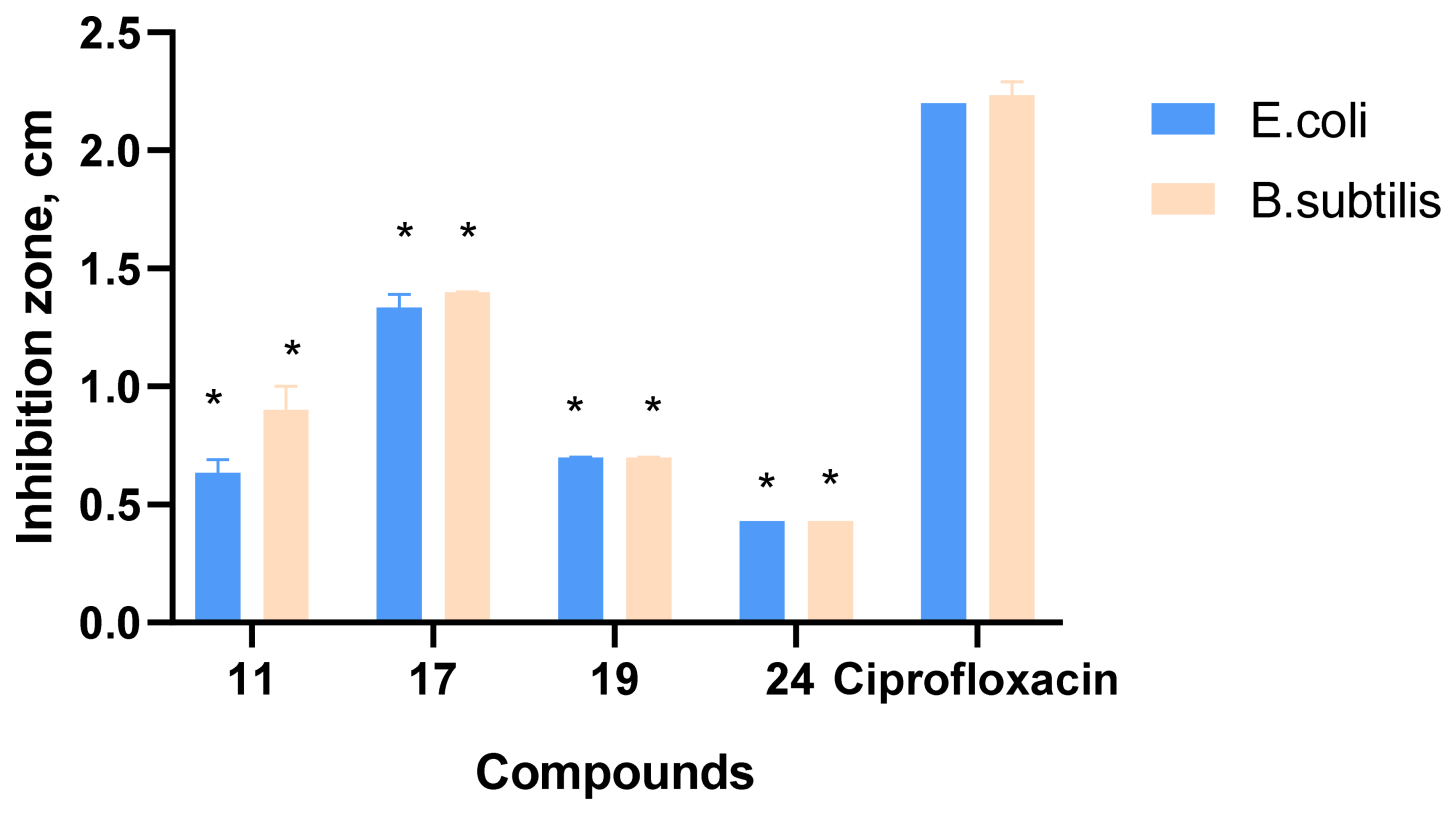
Antibacterial activity of compounds **11**, **17**, **19**, and **24** (20 mM) against *E. coli* and *B. subtilis* (only compounds that were active against bacterial strains are shown). The error bars show the mean ± SD from three replicates. Asterisks show statistically significant comparisons between ciprofloxacin and compounds that were tested with an unpaired two-tailed *t*-test, with a significance level set at * *p* < 0.01.

Screening of antibacterial activity. As seen from the results presented in Figure 7, only four screened compounds exhibited antibacterial activity against *E. coli* and *B. subtilis* bacteria. *N*-(1,3-dioxoisoindolin-2-yl)-3-(phenylamino)propanamide (**17**) was identified as the most active compound (inhibition zones: 1.33 cm ± 0.058 against *E. coli* and 1.4 cm against *B. subtilis*). *N*-(1,3-dioxoisoindolin-2-yl)-3-((4-methoxyphenyl)amino)propanamide (**19**) was shown to possess antibacterial activity against *E. coli* (inhibition zone—0.7 cm) and *B. subtilis* (inhibition zone—0.7 cm). Another compound, propanamide **24** (inhibition zone—0.43 cm against both *E. coli* and *B. subtilis*), is less active, and its activity was surpassed by 3-((4-methoxyphenyl)amino)-*N*’-(4-nitrobenzylidene)propanehydrazide **11** (inhibition zones—0.63 cm ± 0.058 against *E. coli* and 0.9 ± 0.1 cm against *B. subtilis*). Ciprofloxacin was used as a positive control with inhibition zones of 2.2 cm against *E. coli* and 2.23 cm against *B. subtilis*.

## 4. Conclusions

The antioxidant and antibacterial activities of a set of 4-nitrobenzylidene propanehydrazides, phthalimide-containing propanamides, and their corresponding bis-analogues were evaluated using multiple in vitro assays. Overall, monoderivatives frequently exhibited higher activity than the corresponding bis-analogues; however, this trend was not universal, as selected bis-compounds demonstrated notable effects in specific assays (e.g., bis(propanamides) vs. monopropanamides according to FRAP assay; and bis(propanehydrazides vs. monohydrazides according to DPPH assay), indicating that activity depends on both molecular size and compound structure as well as assay type.

Comparative analysis revealed that *N*-(1,3-dioxoisoindolin-2-yl)-3-(unsubstituted and substituted phenylamino)propanamides exhibited stronger antioxidant and antibacterial activity than corresponding 4-nitrobenzylidenepropanehydrazides. Among them, the unsubstituted phenylaminopropanamide **17** displayed the most consistent and pronounced activity across assays, exceeding the reference antioxidant melatonin in both reducing power and DPPH scavenging tests, and showing the highest antibacterial potency. In contrast, within the hydrazone series, phenylaminopropanehydrazide **9** was the most active representative, particularly in the FRAP assay. Among the compounds bearing double sets of pharmacophores, 4-ethoxyphenylamine-based bispropanamide **24** exhibited the highest NBT inhibition, which was 1.58 times higher than that of melatonin.

Structure–activity relationship (SAR) analysis has indicated that the presence of the phthalimide moiety enhances biological activity relative to hydrazone analogues, likely due to improved stability and physicochemical properties; electron-donating substituents on the aromatic ring favour antioxidant activity, following the general trend methoxy ≳ ethoxy > methyl; and excessive structural extension, as in bis-derivatives, does not necessarily improve activity and may reduce it, although specific architectures can retain or enhance particular functions.

In summary, while no single structural motif has been identified to universally be active according to all assays, monoderivative phenylaminopropanamide **17** has emerged as a promising candidate for further development of dual-action antioxidant and antibacterial agents.

## Data Availability

Data is contained within the article or Supplementary Material.

## Supplementary Materials

The following supporting information can be downloaded at: https://www.mdpi.com/article/10.3390/biom16070975/s1, Figures S1–S21: ^1^H NMR, ^13^C NMR, and MS spectra of compounds **9**–**12**, **17**, **18**, and **20**; Figures S22–S30: ^1^H NMR spectra of compounds **13**–**16**, **19**, and **21**–**24**; Tables S1–S7: STDEV values of reducing antioxidant power determination, FRAP, DPPH, ABTS, and NBT assays, and antibacterial screening results; Tables S8–S11: Absorbance values obtained in FRAP, DPPH, ABTS, and NBT assays.

## Abbreviations

The following abbreviations are used in this manuscript:

FRAP	Ferric ion reducing antioxidant power
DPPH	2,2-Diphenyl-1-picrylhydrazyl
ABTS	2,2-azino-bis(3-ethylbenzthiazoline-6-sulfonic acid)
NBT	Nitroblue tetrazolium
ROS	Reactive oxygen species
HAT	hydrogen atom transfer
SET	Single-electron transfer
