# Supplementary material for: Biological Activity and Structure–Activity Relationship of Mono- and Bis-Derivatives of 3-(Arylamino)propanehydrazides
Source: Biomolecules. 2026 Jul 2;16(7):975. doi: 10.3390/biom16070975 (PMC13406754; doi:10.3390/biom16070975)
Supplement: Supplementary file 1 [file biomolecules-16-00975-s001.zip › biomolecules-4368447-supplementary.pdf]

Supplementary Material

Biological Activity and Structure–Activity Relationship of Mono- and Bis-derivatives of 3-(Arylamino)propanehydrazides

Ingrida Tumosienė [1], Ilona Jonuškienė [1,2], Neringa Petrašauskienė [3], and Kristina Kantminienė [3]*

[1]Department of Organic Chemistry, Kaunas University of Technology, Radvilėnų pl. 19, 50254 Kaunas, Lithuania; ingrida.tumosiene@ktu.lt (I.T.); ilona.jonuskiene@ktu.lt (I.J.);

[2] Bioprocess Research Centre, Kaunas University of Technology, Radvilėnų pl. 19, 50254 Kaunas, Lithuania

[3]Department of Physical and Inorganic Chemistry, Kaunas University of Technology, Radvilėnų pl. 19, 50254 Kaunas, Lithuania; neringa.petrasauskiene@ktu.lt (N.P.); kristina.kantminiene@ktu.lt (K.K.)

*Correspondence: kristina.kantminiene@ktu.lt

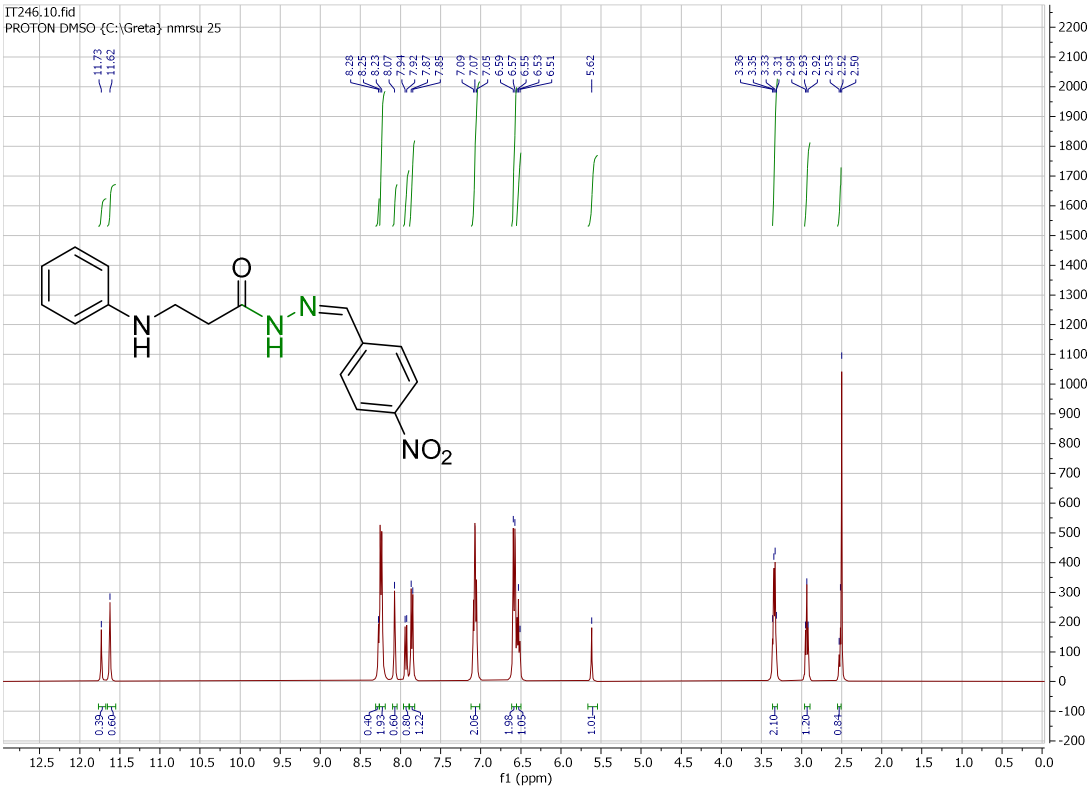

Figure S1. ^{1}H NMR (400 MHz, DMSO-d_6) spectrum of **9**

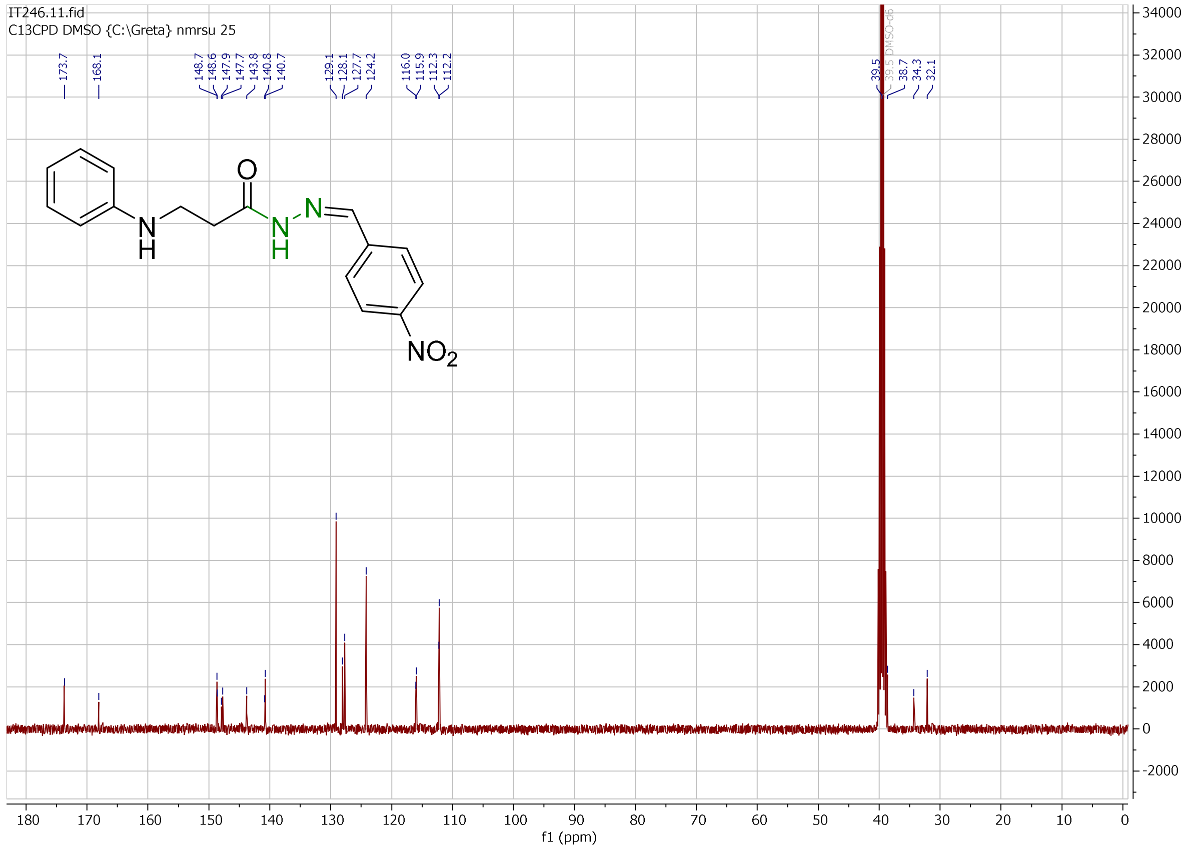

Figure S2. ^{13}C NMR (101 MHz, DMSO-d_6) spectrum of **9**

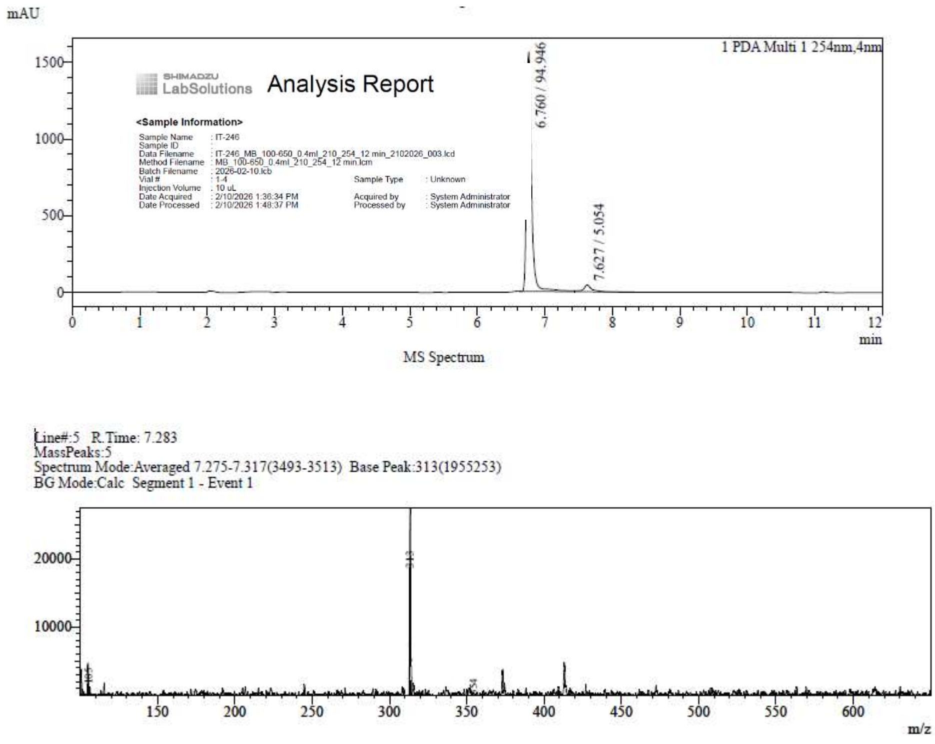

Figure S3. MS spectrum of **9**

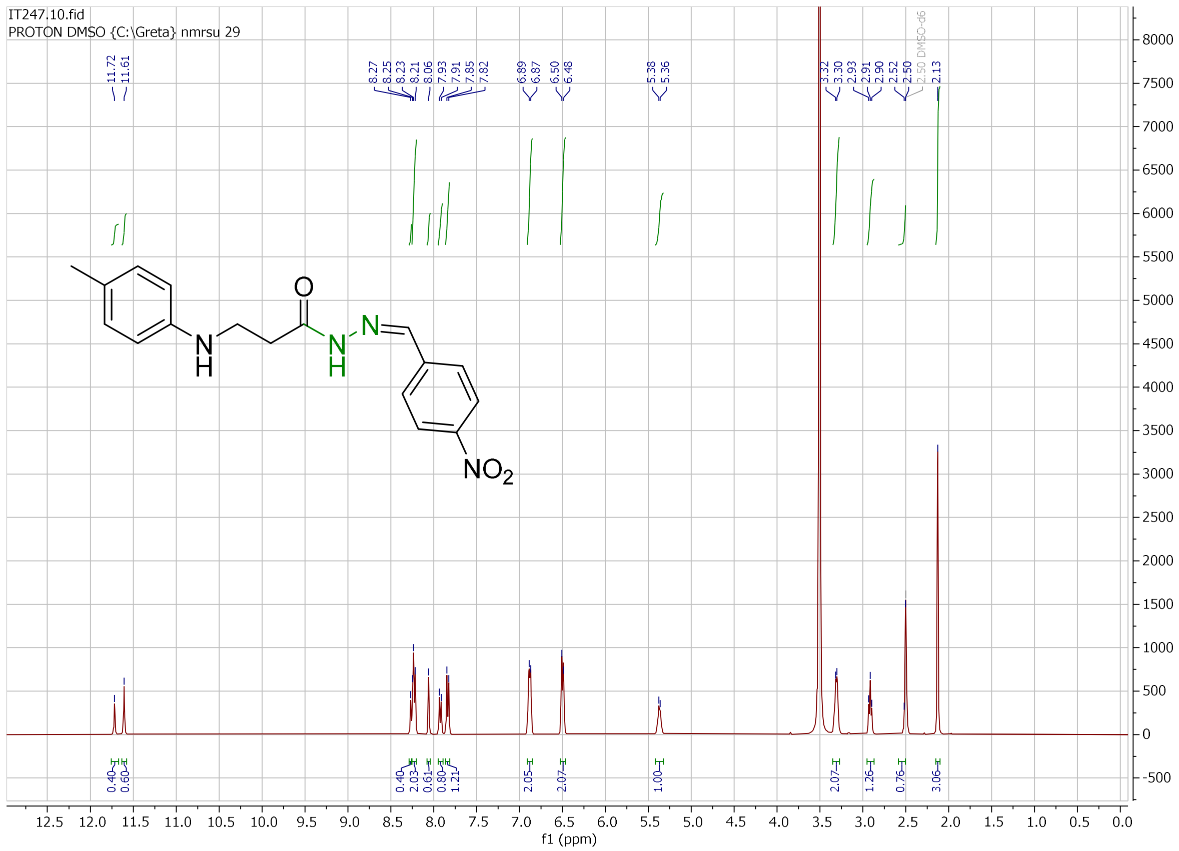

Figure S4. ^{1}H NMR (400 MHz, DMSO-d_6) spectrum of **10**

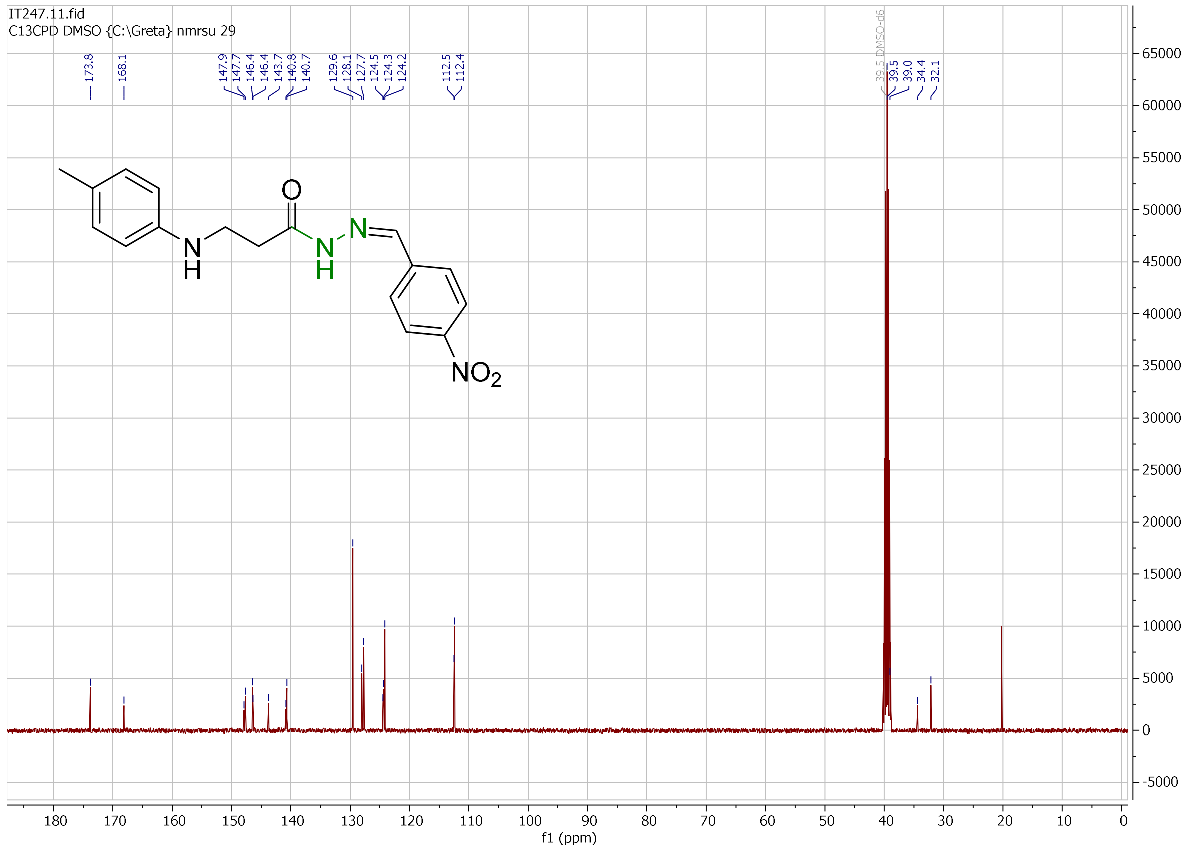

Figure S5. ^{13}C NMR (101 MHz, DMSO-d_6) spectrum of **10**

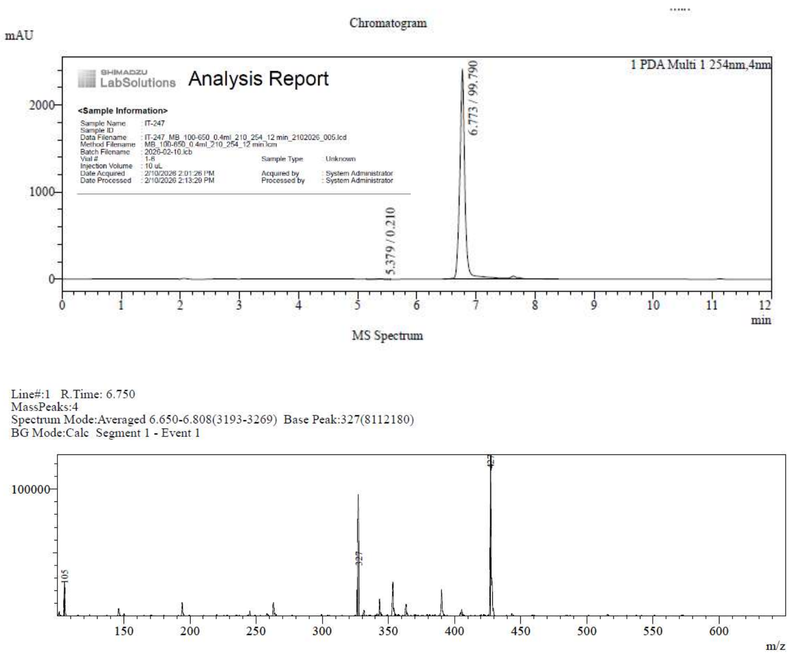

Figure S6. MS spectrum of **10**

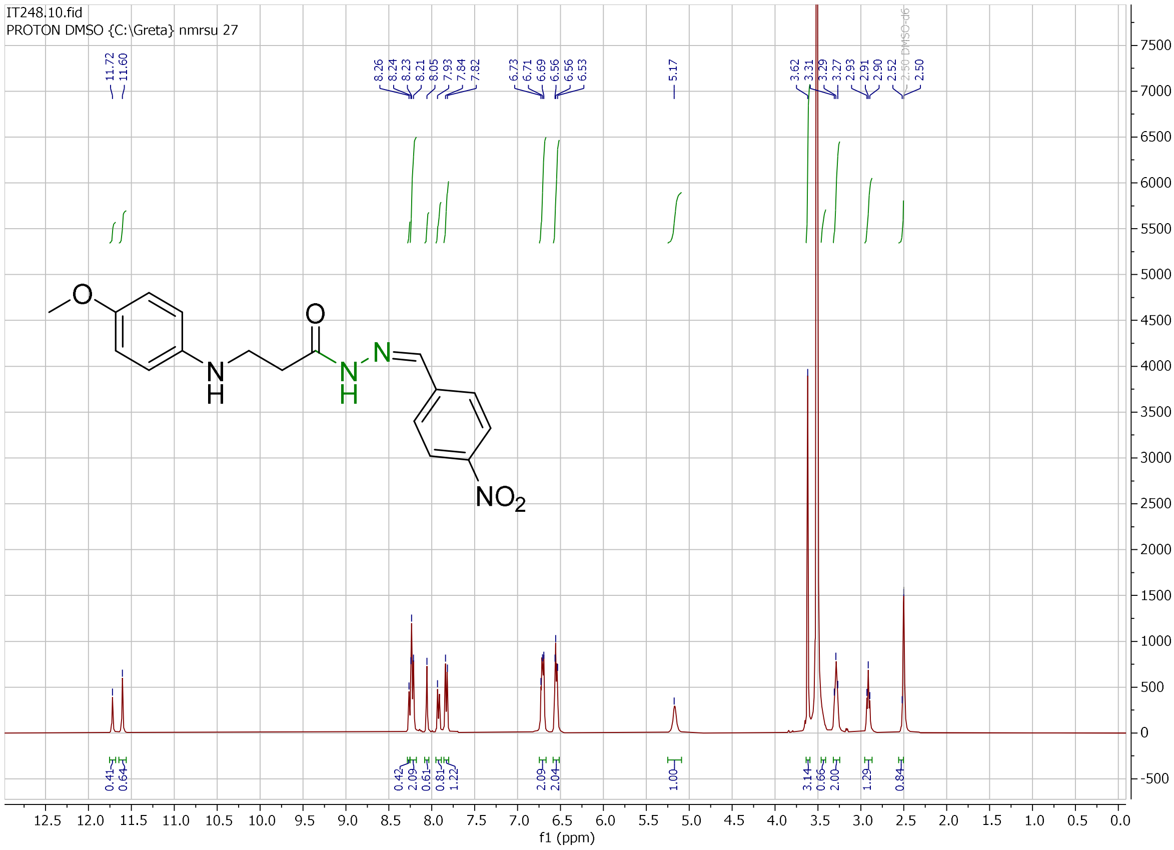

Figure S7. ^{1}H NMR (400 MHz, DMSO-d_6) spectrum of **11**

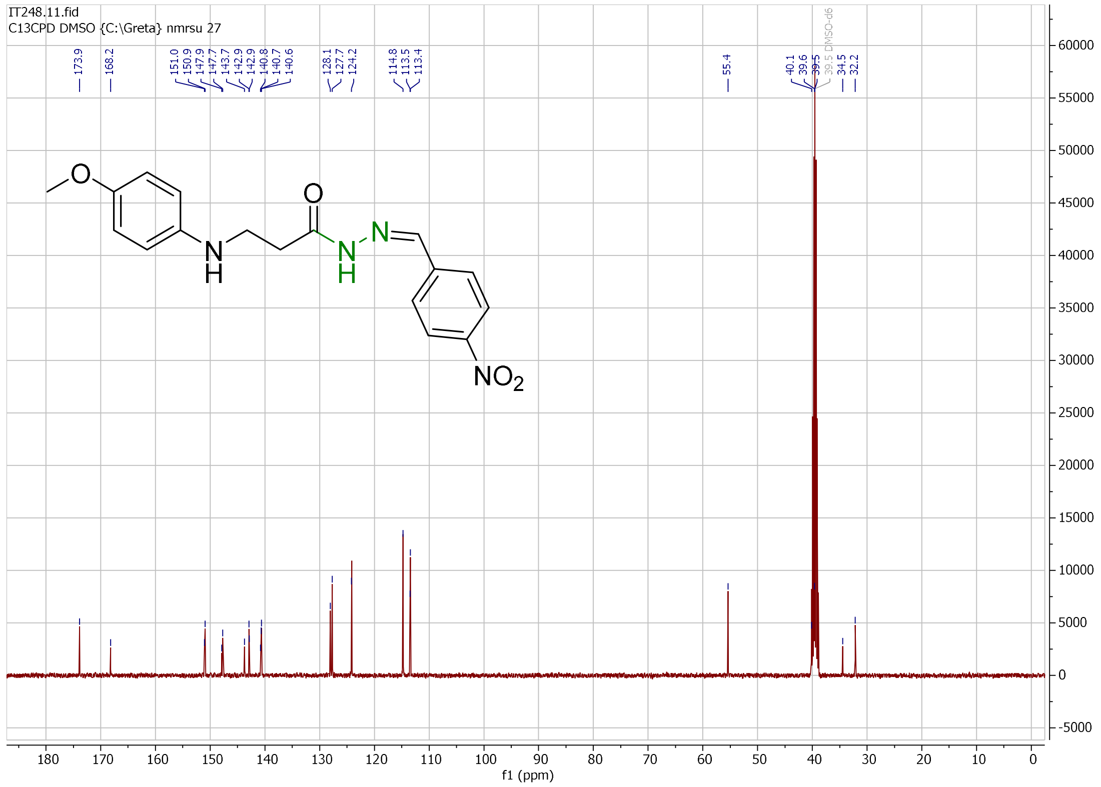

Figure S8. ^{13}C NMR (101 MHz, DMSO-d_6) spectrum of **11**

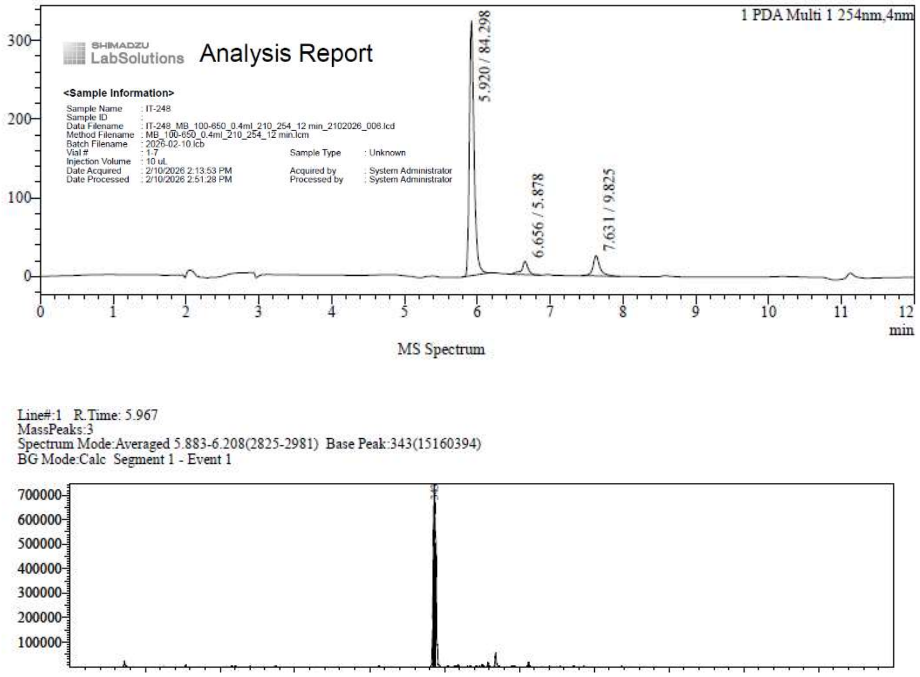

Figure S9. MS spectrum of **11**

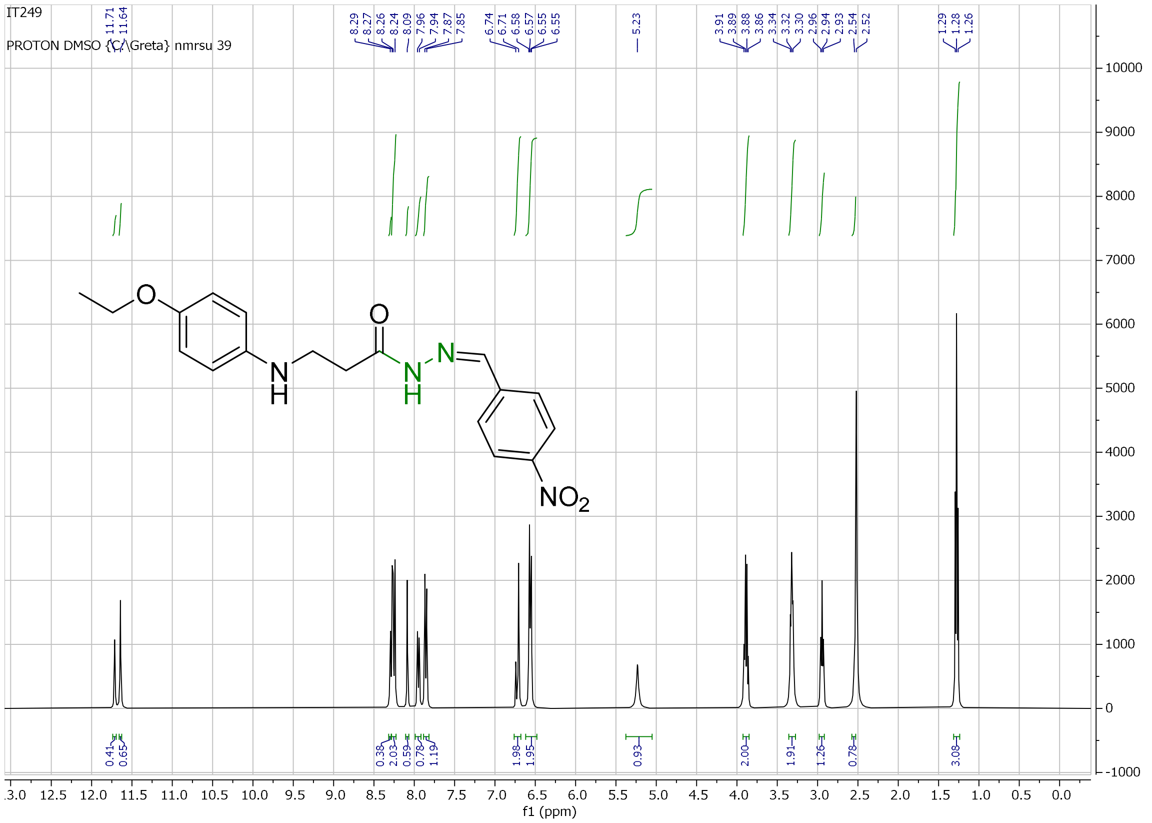

Figure S10. ^{1}H NMR (400 MHz, DMSO-d_6) spectrum of **12**

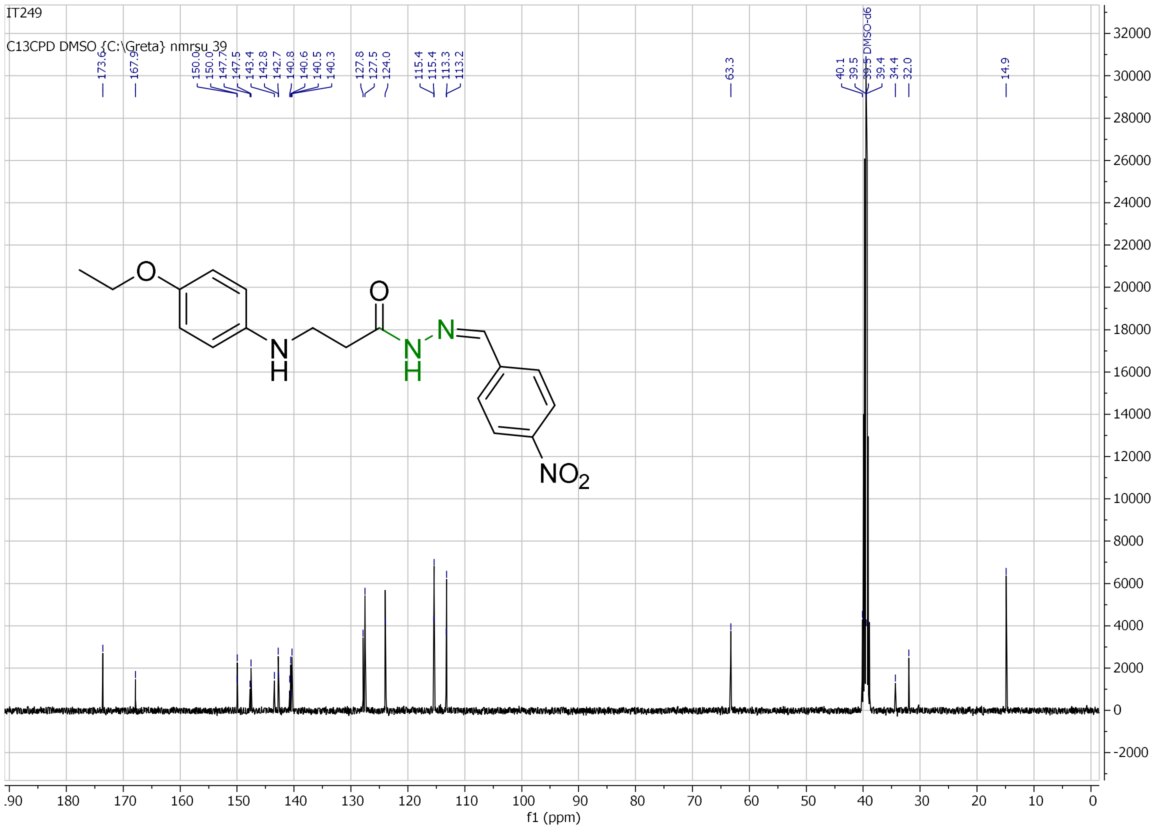

Figure S11. ^{13}C NMR (101 MHz, DMSO-d_6) spectrum of **12**

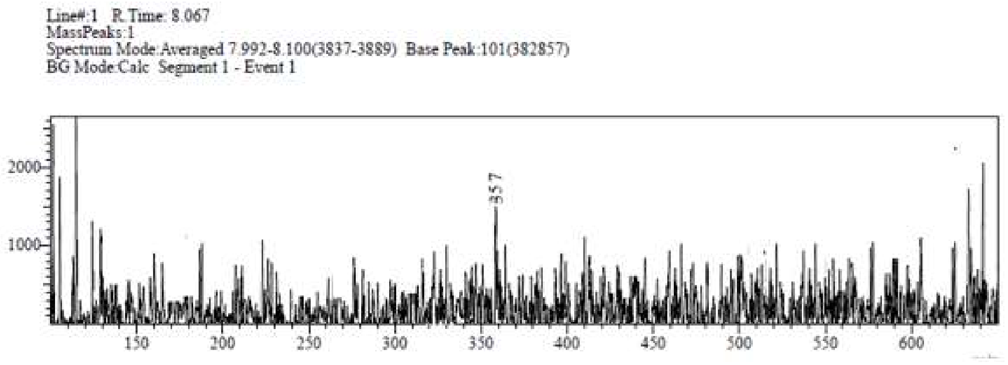

Figure S12. MS spectrum of **12**

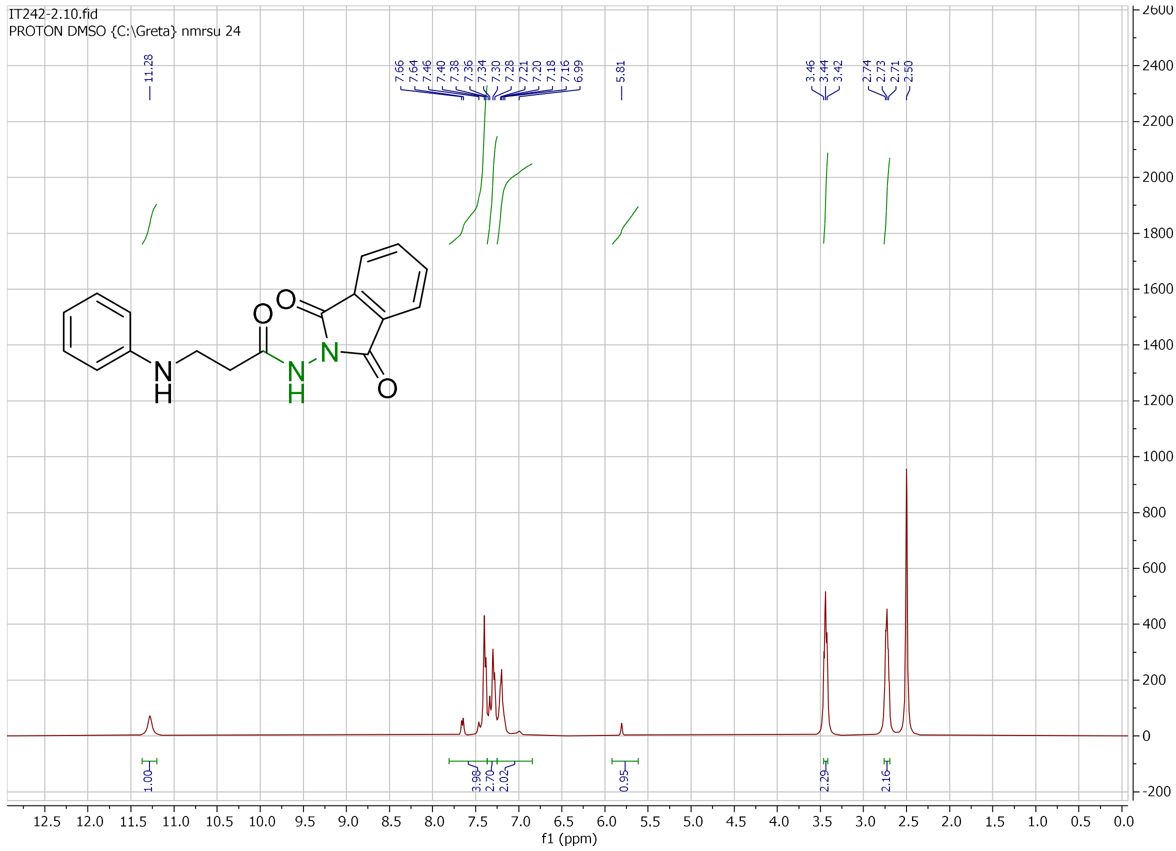

Figure S13. ^{1}H NMR (400 MHz, DMSO-d_6) spectrum of **17**

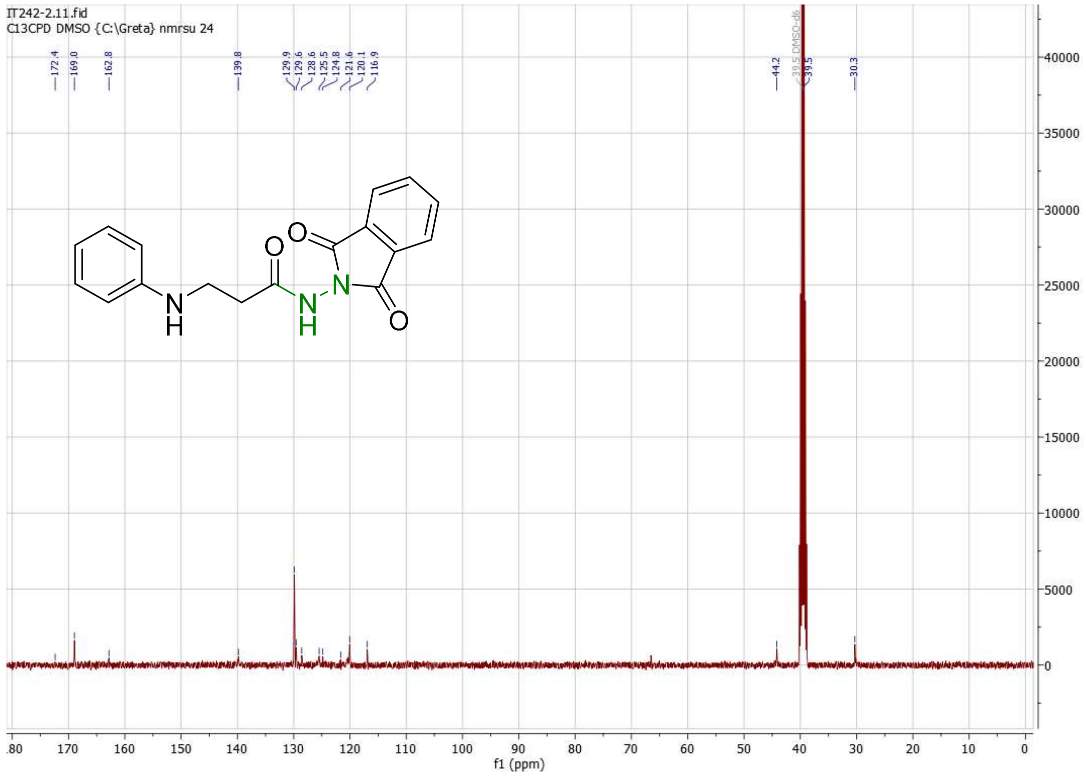

Figure S14. ^{13}C NMR (101 MHz, DMSO-d_6) spectrum of **17**

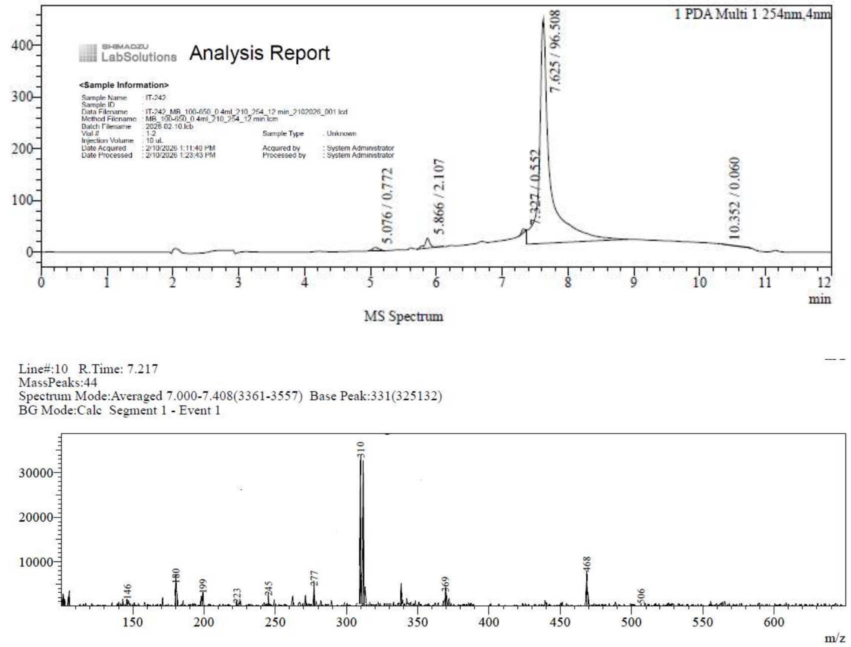

Figure S15. MS spectrum of **17**

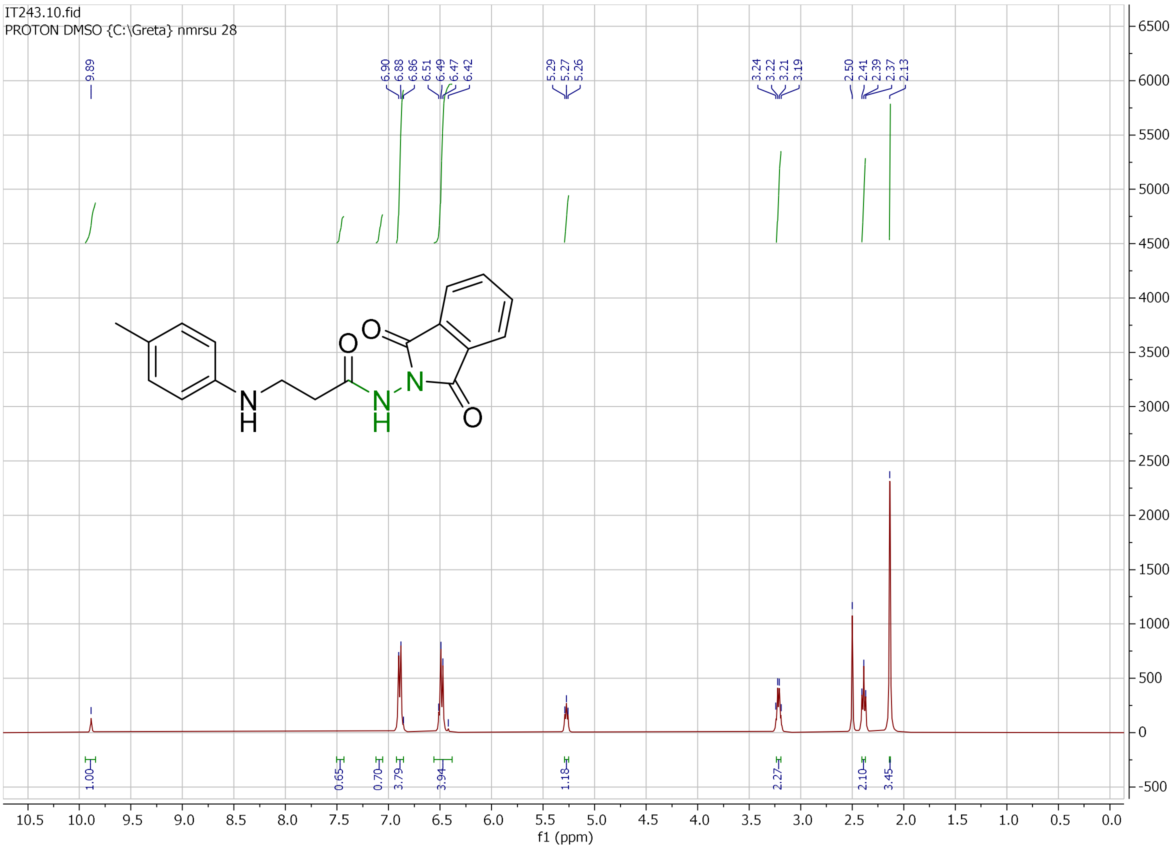

Figure S16. ^{1}H NMR (400 MHz, DMSO-d_6) spectrum of **18**

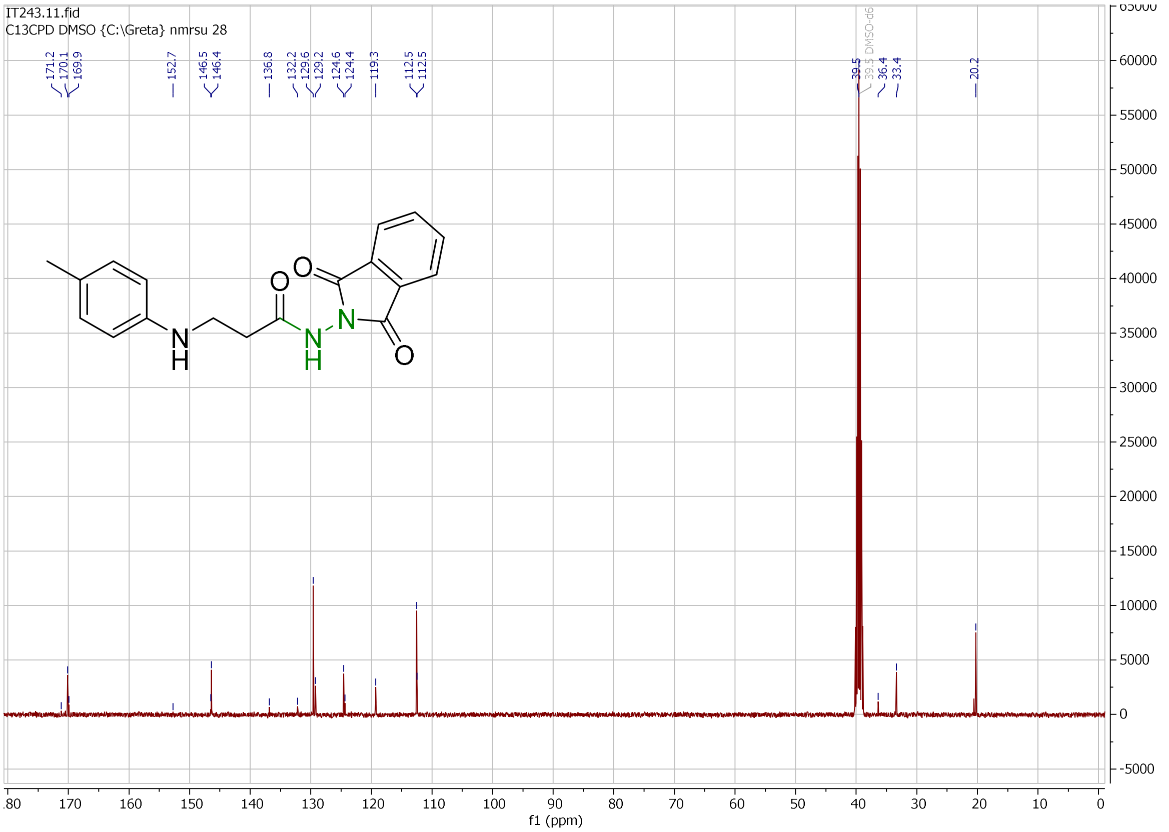

Figure S17. ^{13}C NMR (101 MHz, DMSO-d_6) spectrum of **18**

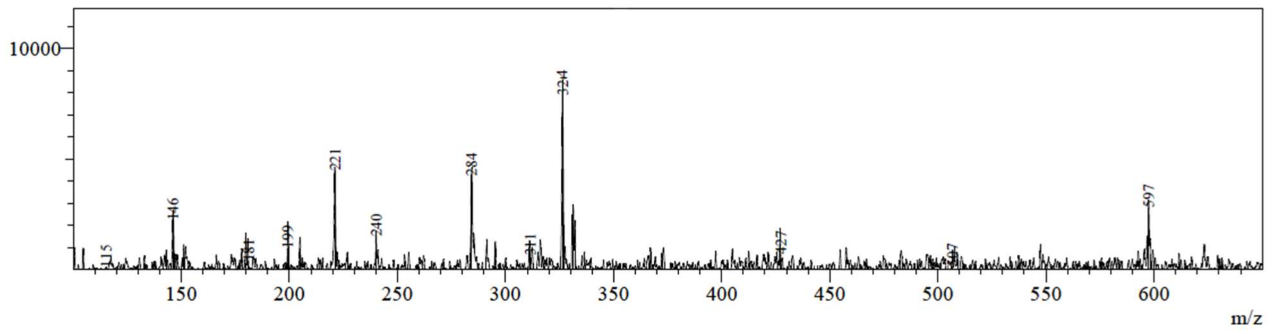

Figure S18. MS spectrum of **18**

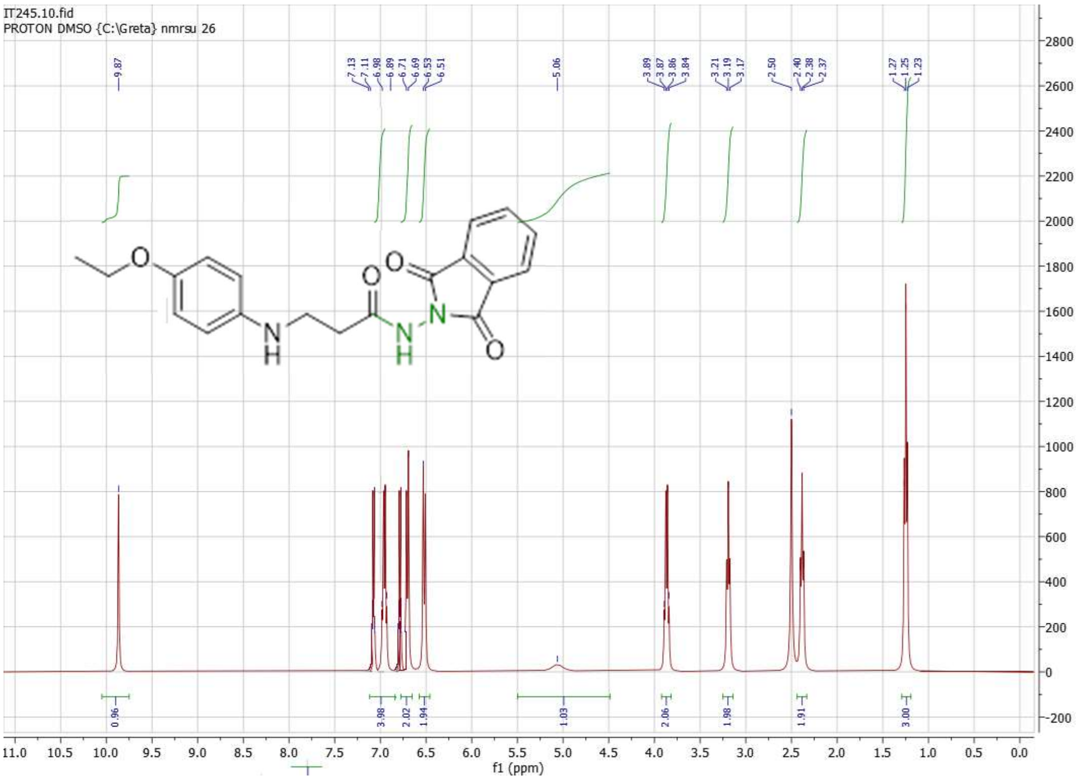

Figure S19. ^{1}H NMR (400 MHz, DMSO-d_6) spectrum of **20**

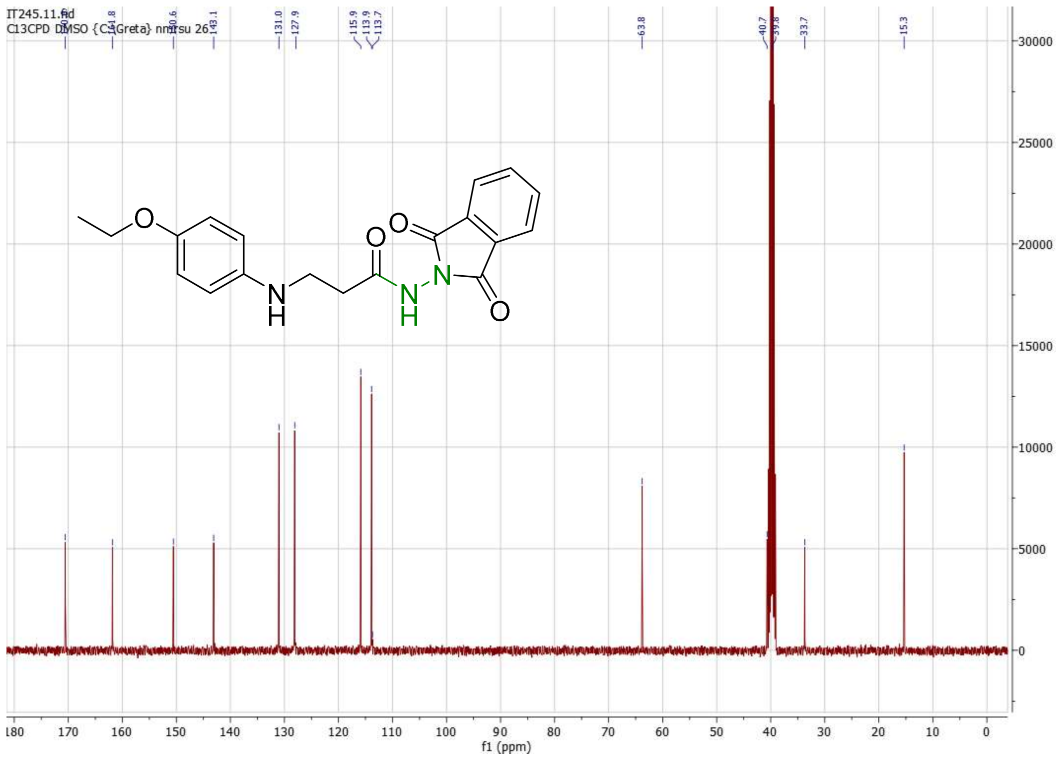

Figure S20. ^{13}C NMR (101 MHz, DMSO-d_6) spectrum of **20**

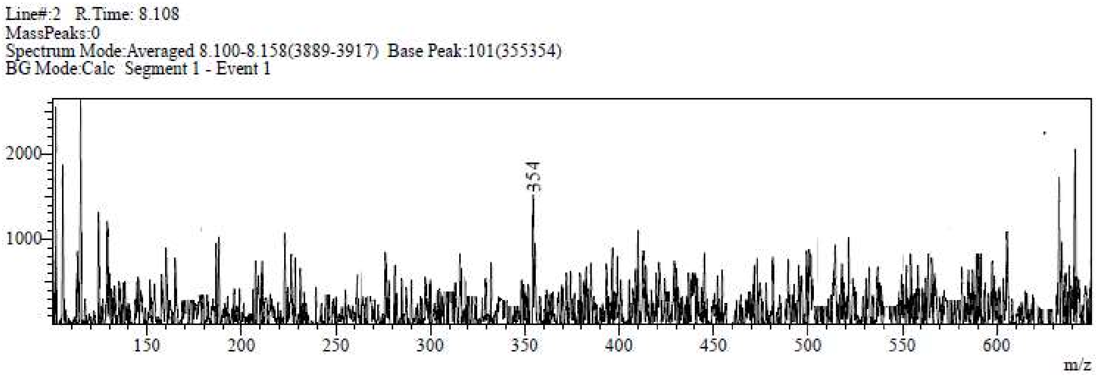

Figure S21. MS spectrum of **20**

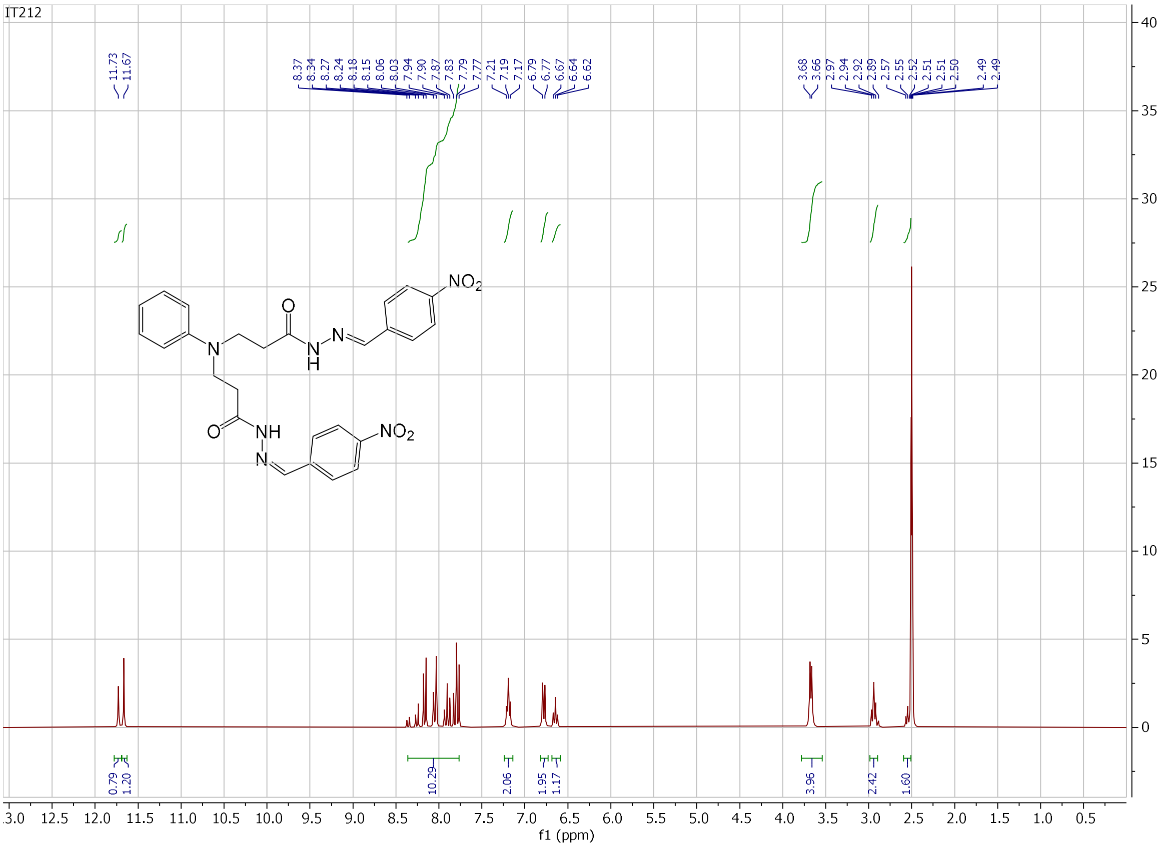

Figure S22. ^{1}H NMR (400 MHz, DMSO-*d*6) spectrum of **13** (originally published in [1])

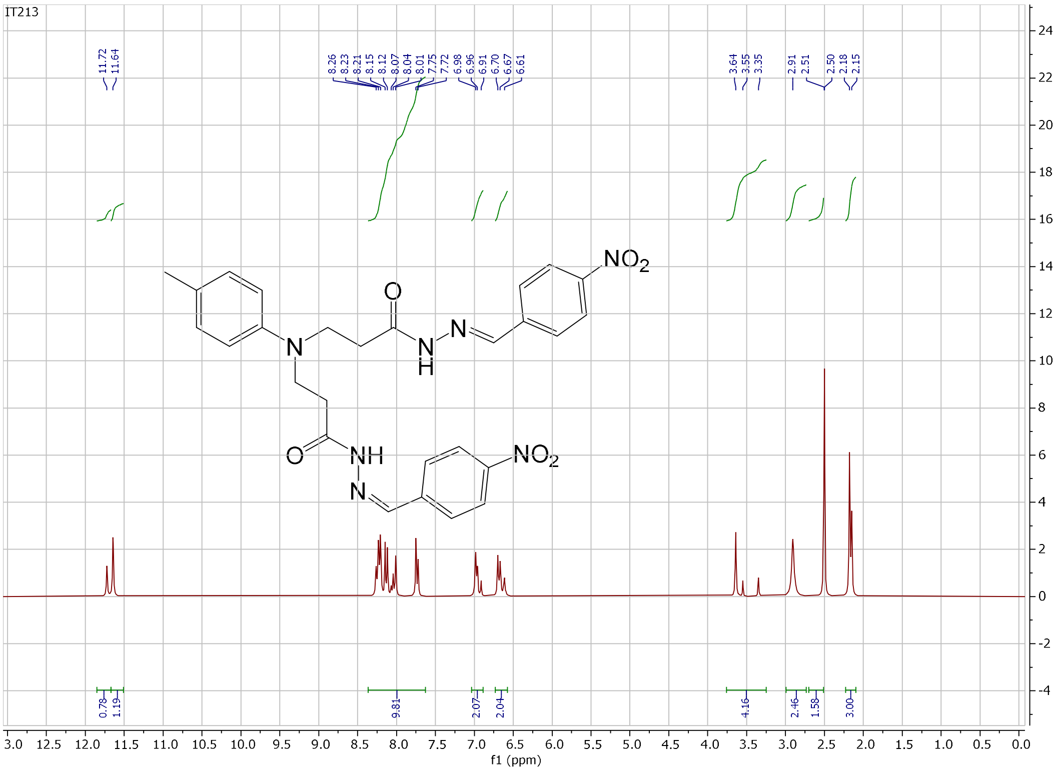

Figure S23. ^{1}H NMR (400 MHz, DMSO-*d*6) spectrum of **14** (originally published in [1])

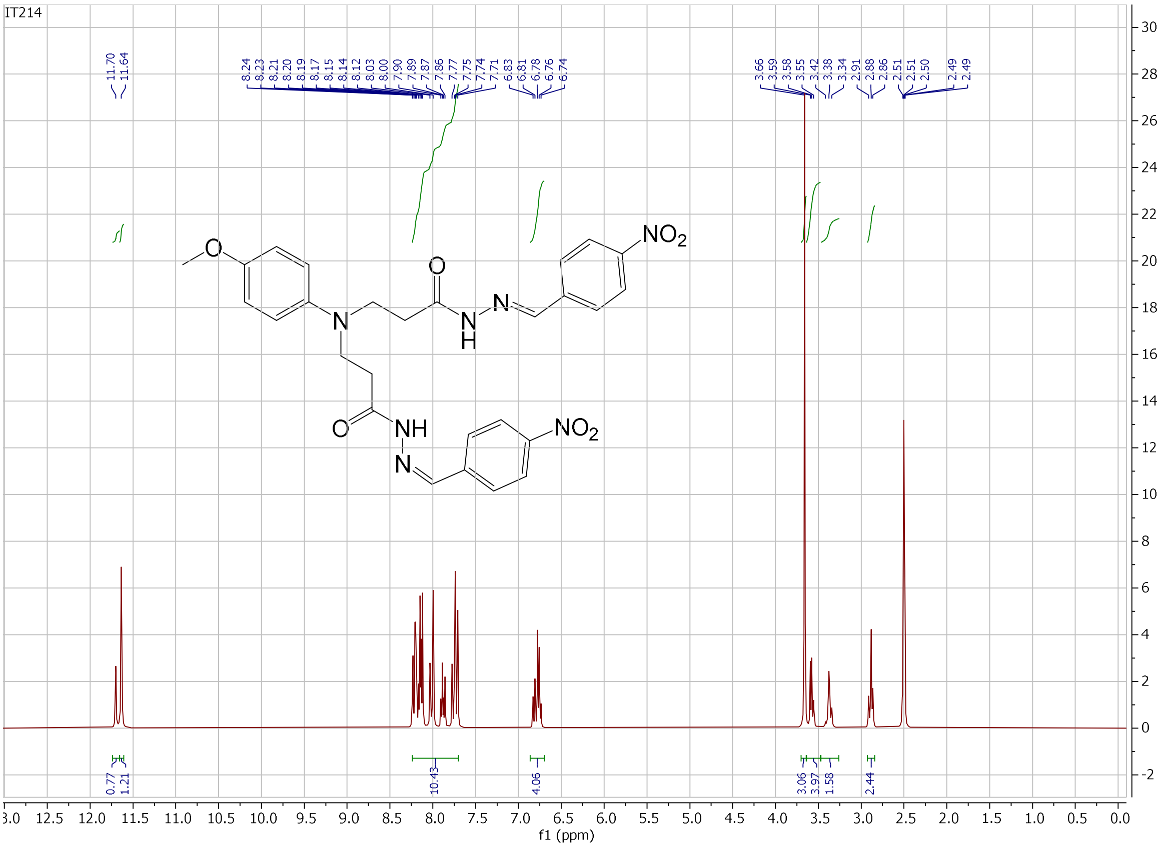

Figure S24. ^{1}H NMR (400 MHz, DMSO-d_6) spectrum of **15** (originally published in [2])

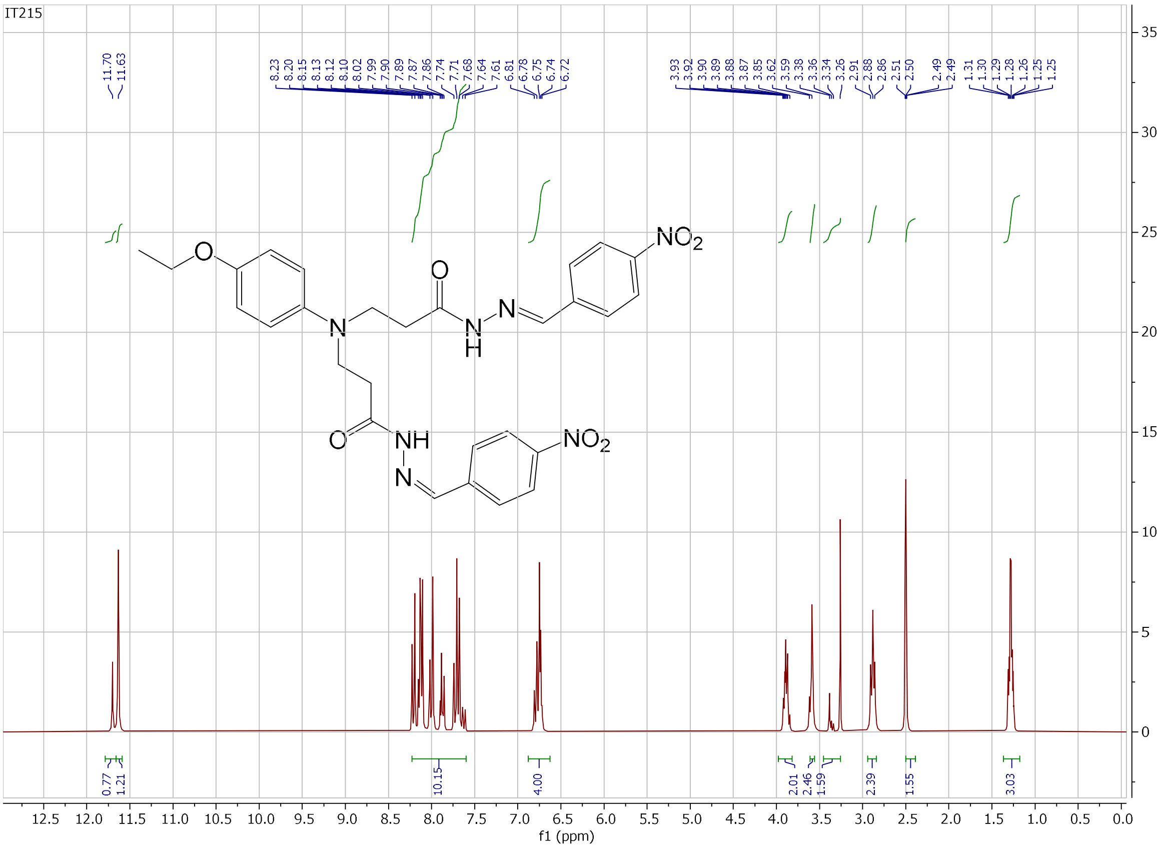

Figure S25. ^{1}H NMR (400 MHz, DMSO-d_6) spectrum of **16** (originally published in [2])

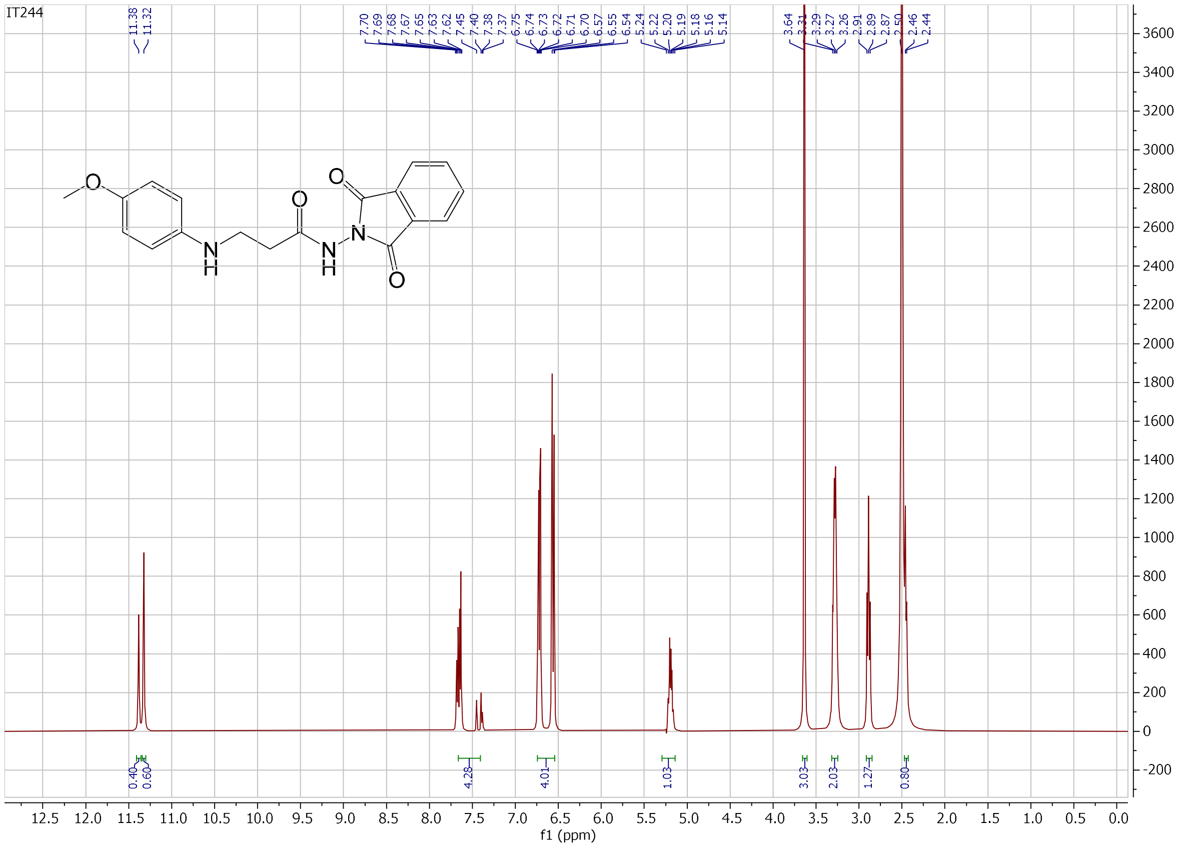

Figure S26. ^{1}H NMR (400 MHz, DMSO-d_6) spectrum of **19** (originally published in [3])

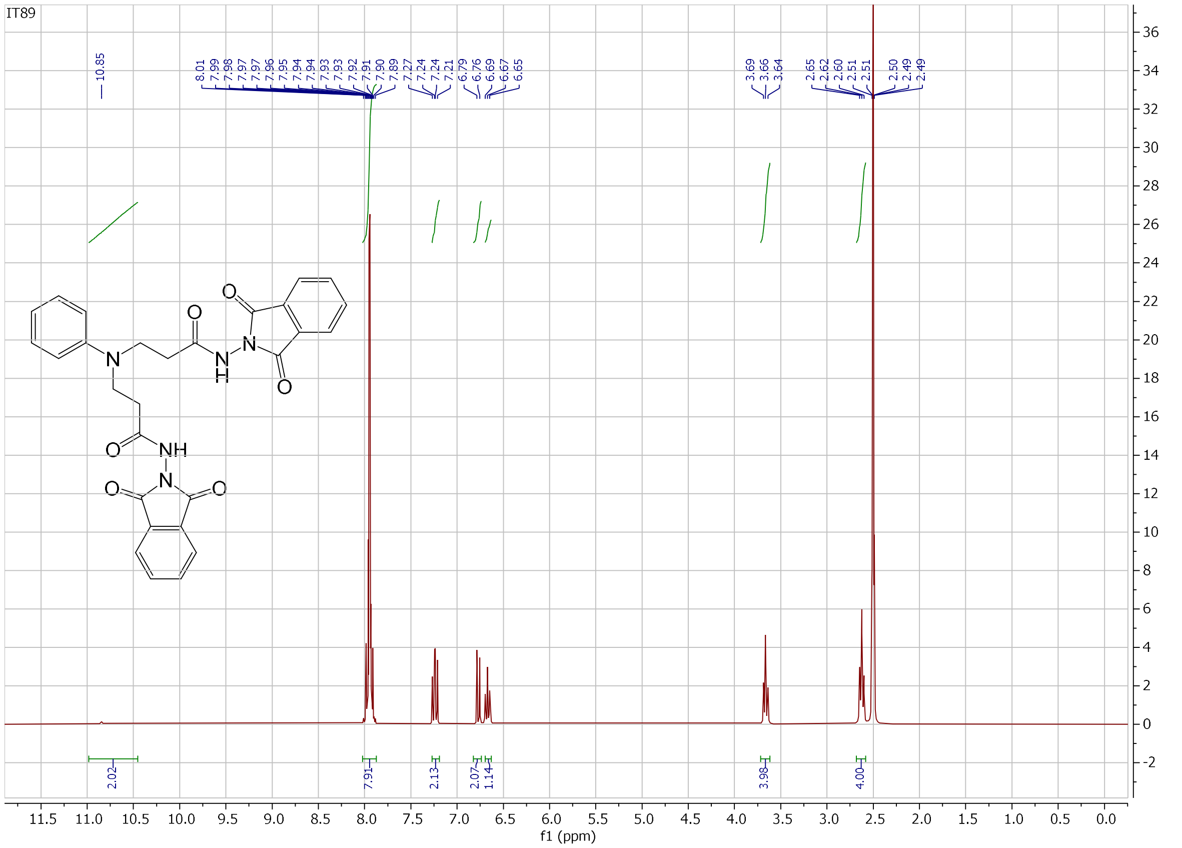

Figure S27. ^{1}H NMR (400 MHz, DMSO-d_6) spectrum of **21** (originally published in [1])

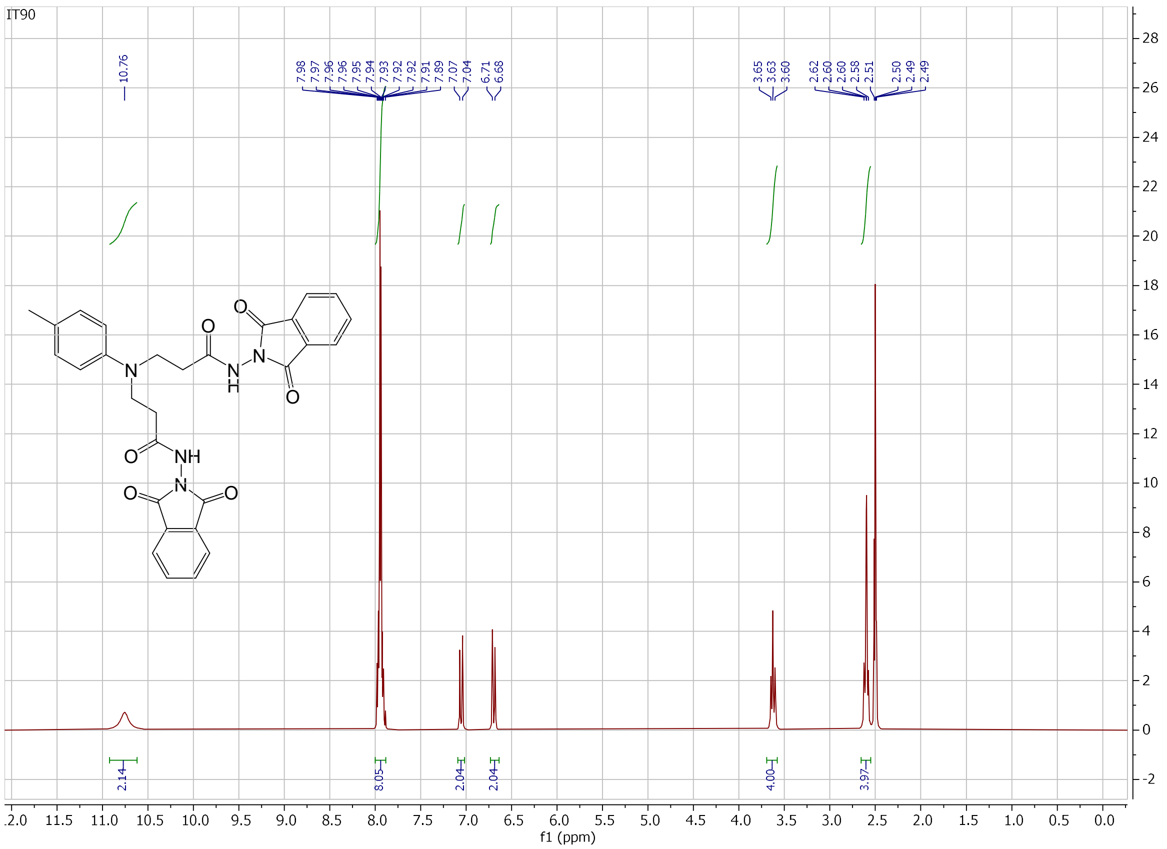

Figure S28. ^{1}H NMR (400 MHz, DMSO-d_6) spectrum of **22** (originally published in [1])

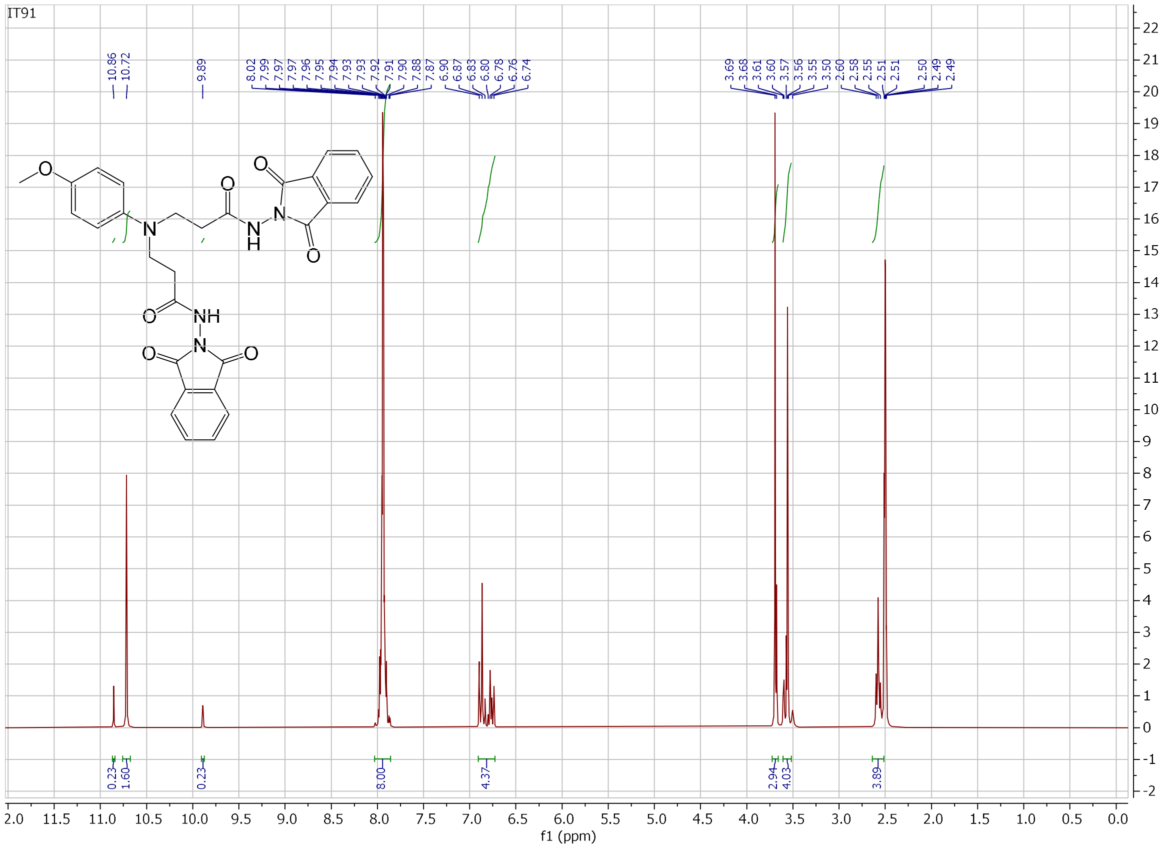

Figure S29. ^{1}H NMR (400 MHz, DMSO-d_6) spectrum of **23** (originally published in [2])

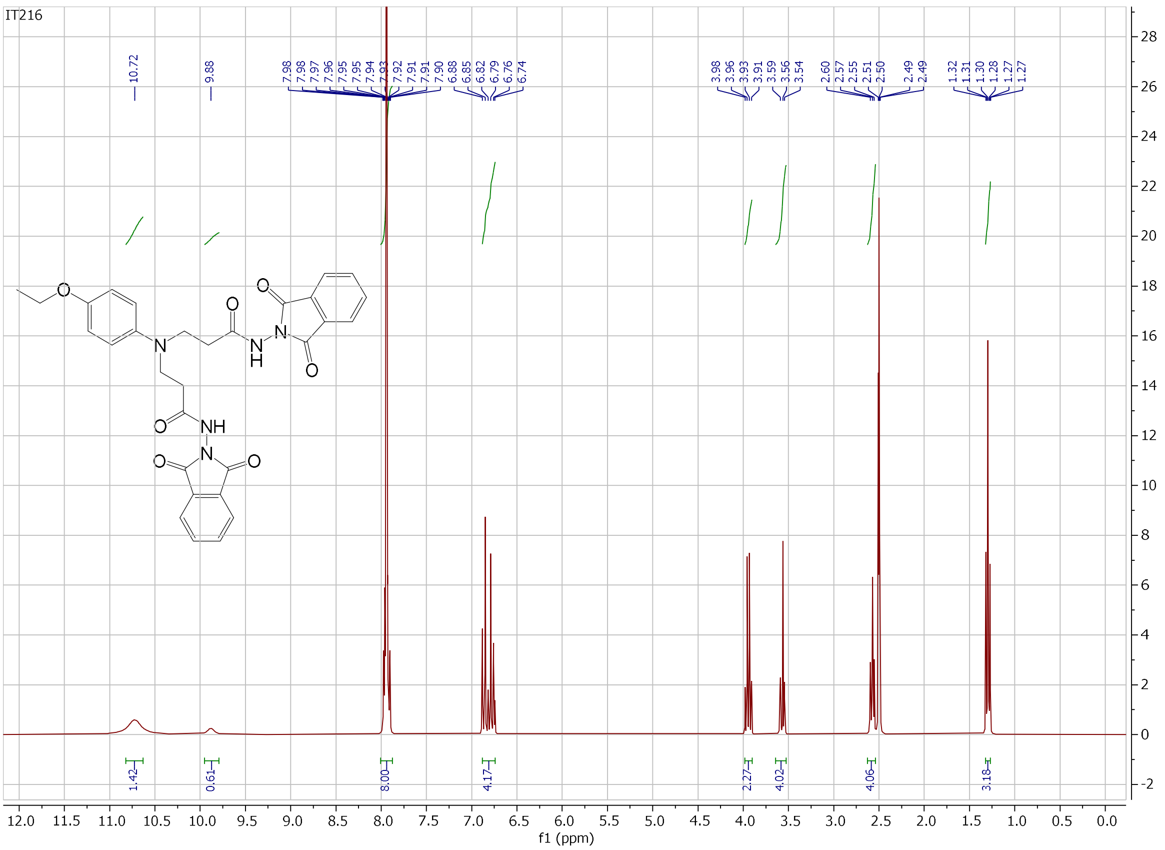

Figure S30. ^{1}H NMR (400 MHz, DMSO-d_6) spectrum of **24** (originally published in [2])

Table S1. STDEV values of Ferric ion (Fe^{3+}) reducing antioxidant power determination assay

Compound	1	2	3	AVG	STDEV
**Melatonin**	1.165	1.182	1.204	1.184	0.020
**9**	0.657	0.673	0.688	0.673	0.016
**10**	0.16	0.164	0.162	0.162	0.002
**11**	1.852	1.943	1.912	1.902	0.046
**12**	1.727	1.795	1.815	1.779	0.046
**13**	0.048	0.053	0.074	0.058	0.014
**14**	0.332	0.369	0.381	0.361	0.026
**15**	0.09	0.092	0.097	0.093	0.004
**16**	0.035	0.04	0.041	0.039	0.003
**17**	1.99	2.001	2.024	2.005	0.017
**18**	0.77	0.822	0.863	0.818	0.047
**19**	0.68	0.684	0.68	0.681	0.002
**20**	1.854	1.854	1.841	1.850	0.008
**21**	0.159	0.161	0.16	0.16	0.001
**22**	0.416	0.425	0.443	0.428	0.014
**23**	0.935	0.9549	0.965	0.952	0.015
**24**	0.612	0.618	0.676	0.635	0.035

Table S2. STDEV values of ferric reducing antioxidant power (FRAP) assay

Compound	1	2	3	AVG	STDEV
**Melatonin**	69.342	67.699	68.169	68.404	0.846
**9**	100.563	101.972	100.141	100.892	0.959
**10**	90.845	91.0323	91.174	91.017	0.165
**11**	93.145	92.629	92.817	92.864	0.261
**12**	95.023	95.446	95.211	95.227	0.212
**13**	16.619	16.948	16.808	16.792	0.165
**14**	90.610	91.174	92.300	91.362	0.861
**15**	78.216	78.404	78.779	78.466	0.287
**16**	75.399	76.526	77.512	76.479	1.057
**17**	90.328	90	90.469	90.266	0.241
**18**	89.718	89.671	89.718	89.703	0.0271
**19**	65.506	65.51	65.52	65.512	0.007
**20**	89.859	90.00	90.141	90.00	0.141
**21**	96.197	93.756	93.568	94.507	1.466
**22**	91.127	91.268	91.643	91.346	0.267
**23**	98.028	96.808	95.352	96.729	1.340
**24**	90.563	90.141	90.282	90.329	0.215

Table S3. STDEV values of 1,1-diphenyl-2-picrylhydrazyl (DPPH) radical scavenging assay

Compound	1	2	3	AVG	STDEV
**Melatonin**	13.44	13.44	13.12	13.33	0.185
**9**	30.5	29.7	30.02	30.07	0.403
**10**	37.35	36.56	36.88	36.93	0.397
**11**	34.48	34.8	34.64	34.64	0.160
**12**	37.67	37.51	37.51	37.56	0.092
**13**	23.33	22.69	20.78	22.27	1.327
**14**	63.34	63.18	58.4	61.64	2.807
**15**	0.85	4.99	5.95	3.93	2.710
**17**	89.48	89.48	89.32	89.43	0.092
**18**	4.2	4.36	4.52	4.36	0.160
**19**	79.63	79.62	79.62	79.62	0.006
**20**	45.32	45.64	45.8	45.59	0.244
**21**	9.3	9.94	9.78	9.67	0.333
**22**	12.49	10.41	8.82	10.57	1.840
**23**	10.1	10.26	10.57	10.31	0.239
**24**	88.36	88.36	88.2	88.31	0.092

Table S4. STDEV values of 2,2'-azino-bis(3-ethylbenzothiazoline-6-sulfonic acid (ABTS) assay

Compound	1	2	3	AVG	STDEV
**Melatonin**	100	100	100	100	0
**9**	100	100	100	100	0
**10**	100	100	100	100	0
**11**	98.87	99.35	99.11	99.11	0.24
**12**	90.62	89.43	87.17	89.07	1.75
**13**	97.68	98.81	98.22	98.24	0.57
**14**	98.87	98.75	98.63	98.75	0.12
**15**	99.76	98.4	99.17	99.11	0.68
**16**	98.28	98.52	98.81	98.54	0.27
**17**	100	100	100	100	0
**18**	99.05	98.75	98.4	98.73	0.33
**19**	96.18	96.19	96.19	96.19	0.01
**20**	100	100	100	100	0
**21**	96.5	94.42	95.49	95.47	1.04
**22**	97.09	98.87	98.52	98.16	0.94
**23**	100	99.58	99.7	99.76	0.22
**24**	99.17	99.28	98.93	99.13	0.18

Table S5. STDEV values of the nitroblue tetrazolium (NBT) reduction method

Compound	1	2	3	AVG	STDEV
**Melatonin**	56.89	52.17	54.23	54.43	2.368
**9**	66.63	53.35	50.98	56.99	8.435
**10**	28.25	33.86	46.85	36.32	9.541
**11**	72.24	76.38	71.65	73.42	2.577
**12**	23.52	16.14	20.28	19.98	3.699
**14**	65.75	61.02	70.77	65.85	4.876
**15**	67.81	79.04	71.65	72.83	5.707
**16**	61.02	58.96	60.14	60.04	1.033
**19**	25.2	25.00	25.3	25.17	0.153
**21**	39.17	40.06	39.76	39.66	0.453
**22**	42.42	41.83	38.88	41.04	1.897
**23**	71.95	64.57	71.36	69.29	4.101
**24**	86.71	84.94	86.42	86.02	0.949

Table S6. STDEV values of antibacterial activity of compounds against *E. coli*

Compound	1	2	3	AVG	STDEV
**11**	0.6	0.7	0.6	0.63	0.057
**17**	1.3	1.4	1.3	1.33	0.0577
**19**	0.7	0.7	0.7	0.7	0
**24**	0.43	0.43	0.43	0.43	0
**Ciprofloxacin**	2.2	2.2	2.2	2.2	0

Table S7. STDEV values of antibacterial activity of compounds against *B. subtilis*

Compound	1	2	3	AVG	STDEV
**11**	1	0.9	0.8	0.9	0.1
**17**	1.4	1.4	1.4	1.4	0
**19**	0.7	0.7	0.7	0.7	0
**24**	0.43	0.43	0.43	0.43	0
**Ciprofloxacin**	2.2	2.2	2.3	2.23	0.057

Table S8. Absorbance values of ferric reducing antioxidant power (FRAP) assay

	1	2	3
**Melatonin**	1.477	1.442	1.452
**9**	2.142	2.172	2.133
**10**	1.935	1.939	1.942
**11**	1.984	1.973	1.977
**12**	2.024	2.033	2.028
**13**	0.354	0.361	0.358
**14**	1.93	1.942	1.966
**15**	1.666	1.67	1.678
**16**	1.606	1.63	1.651
**17**	1.924	1.917	1.927
**18**	1.911	1.91	1.911
**19**	1.395	1.395	1.395
**20**	1.914	1.917	1.92
**21**	2.049	1.997	1.993
**22**	1.941	1.944	1.952
**23**	2.088	2.062	2.031
**24**	1.929	1.92	1.923

Table S9. Absorbance values of 1,1-diphenyl-2-picrylhydrazyl (DPPH) radical scavenging assay

	1	2	3
**Control**	0.636	0.628	0.618
**Melatonin**	0.543	0.543	0.545
**9**	0.436	0.441	0.439
**10**	0.393	0.398	0.396
**11**	0.411	0.409	0.41
**12**	0.391	0.392	0.392
**13**	0.481	0.485	0.497
**14**	0.23	0.231	0.261
**15**	0.622	0.596	0.59
**16**	0	0	0
**17**	0.066	0.066	0.067
**18**	0.601	0.6	0.599
**19**	0.1278	0.12785	0.12785
**20**	0.343	0.341	0.34
**21**	0.569	0.565	0.566
**22**	0.549	0.562	0.572
**23**	0.564	0.563	0.561
**24**	0.073	0.073	0.074

Table S10. Absorbance values of 2,2'-azino-bis(3-ethylbenzothiazoline-6-sulfonic acid (ABTS) assay

	1	2	3
**Control**	1.684	1.684	1.684
**Melatonin**	0	0	0
**9**	0	0	0
**10**	0	0	0
**11**	0.019	0.011	0.015
**12**	0.158	0.178	0.216
**13**	0.039	0.02	0.03
**14**	0.019	0.021	0.023
**15**	0.004	0.027	0.014
**16**	0.029	0.025	0.02
**17**	0	0	0
**18**	0.016	0.021	0.027
**19**	0.0642	0.0641	0.0641
**20**	0	0	0
**21**	0.059	0.094	0.076
**22**	0.049	0.019	0.025
**23**	0	0.007	0.005
**24**	0.014	0.012	0.018

Table S11. Absorbance values of the nitroblue tetrazolium (NBT) reduction method

	1	2	3
**Control**	0.341	0.35	0.325
**Melatonin**	0.146	0.162	0.155
**9**	0.113	0.158	0.166
**10**	0.243	0.224	0.18
**11**	0.094	0.08	0.096
**12**	0.259	0.284	0.27
**13**	0.335	0.355	0.342
**14**	0.116	0.132	0.099
**15**	0.109	0.071	0.096
**16**	0.132	0.139	0.135
**17**	0.336	0.34	0.343
**18**	0.396	0.422	0.38
**19**	0.2533	0.254	0.253
**20**	0.377	0.416	0.423
**21**	0.206	0.203	0.204
**22**	0.195	0.197	0.207
**23**	0.095	0.12	0.097
**24**	0.045	0.051	0.046

References

1. Tumosiene, I.; Jakiene, E.; Beresnevicius, Z.J.; Mikulskiene, G. Synthesis and Properties of Dihydrazides of N-Phenyl-and N-(4-Methylphenyl)-N-Carboxyethyl-β-Alanines. *Cheminė Technol.* **2006**, *3*, 58–64.
2. Tumosienė, I.; Mikulskiene, G.; Kantminiene, K.; Beresnevičius, Z.J. Synthesis and Structure of 3,3′-[(4-Alkoxyphenyl)Imino] Bis(N′-Phthaloyl- or N′-Benzylidenepropanohydrazide) Derivatives. *Chemija* **2011**, *22*, 65–72.
3. Tumosienė, I.; Kantminienė, K.; Klevinskas, A.; Petrikaitė, V.; Jonuškienė, I.; Mickevičius, V. Antioxidant and Anticancer Activity of Novel Derivatives of 3-[(4-Methoxyphenyl)Amino]Propanehydrazide. *Molecules* **2020**, *25*, doi:10.3390/molecules25132980.
